# Cooperative and Antagonistic Contributions of Two Heterochromatin Proteins to Transcriptional Regulation of the *Drosophila* Sex Determination Decision

**DOI:** 10.1371/journal.pgen.1002122

**Published:** 2011-06-09

**Authors:** Hui Li, Janel Rodriguez, Youngdong Yoo, Momin Mohammed Shareef, RamaKrishna Badugu, Jamila I. Horabin, Rebecca Kellum

**Affiliations:** 1 Department of Biology, University of Kentucky, Lexington, Kentucky, United States of America; 2 Department of Biomedical Sciences, Florida State University, Tallahassee, Florida, United States of America; Max-Planck-Institute of Immunobiology, Germany

## Abstract

Eukaryotic nuclei contain regions of differentially staining chromatin (heterochromatin), which remain condensed throughout the cell cycle and are largely transcriptionally silent. RNAi knockdown of the highly conserved heterochromatin protein HP1 in *Drosophila* was previously shown to preferentially reduce male viability. Here we report a similar phenotype for the telomeric partner of HP1, HOAP, and roles for both proteins in regulating the *Drosophila* sex determination pathway. Specifically, these proteins regulate the critical decision in this pathway, firing of the establishment promoter of the masterswitch gene, *Sex-lethal (Sxl)*. Female-specific activation of this promoter, *Sxl_Pe_*, is essential to females, as it provides SXL protein to initiate the productive female-specific splicing of later *Sxl* transcripts, which are transcribed from the maintenance promoter (*Sxl_Pm_*) in both sexes. HOAP mutants show inappropriate *Sxl_Pe_* firing in males and the concomitant inappropriate splicing of *Sxl_Pm_*-derived transcripts, while females show premature firing of *Sxl_Pe_*. HP1 mutants, by contrast, display *Sxl_Pm_* splicing defects in both sexes. Chromatin immunoprecipitation assays show both proteins are associated with *Sxl_Pe_* sequences. In embryos from HP1 mutant mothers and *Sxl* mutant fathers, female viability and RNA polymerase II recruitment to *Sxl_Pe_* are severely compromised. Our genetic and biochemical assays indicate a repressing activity for HOAP and both activating and repressing roles for HP1 at *Sxl_Pe_*.

## Author Summary

Eukaryotic genomes are organized into two distinct classes of chromatin, euchromatin and heterochromatin. The former is less condensed to enable transcription, whereas heterochromatin, which is marked by Heterochromatin Protein 1 (HP1), remains compact and mostly transcriptionally silent throughout the cell cycle. The viability of *Drosophila* males is known to be preferentially compromised in mutants for HP1 and some HP1-associated proteins, suggesting more generalized roles for these proteins in sex-specific gene expression. We now describe a male viability defect for the telomeric partner of HP1, HOAP, and misregulation of the sex determination pathway. Key to the sex determination process is the activation of the X chromosome dose sensing promoter of *Sex-lethal*, *Sxl_Pe_*. We provide genetic and biochemical evidence that HOAP, the telomere binding partner of HP1, has repressing activity; while HP1 has both activating and repressing roles at this critical promoter. Chromatin immunoprecipitation assays show both proteins are associated with *Sxl_Pe_* sequences. Additionally, RNA polymerase II association with *Sxl_Pe_* shows a requirement for HP1, suggesting a transcription initiation role for HP1. Combined, our data implicate HP1 and HOAP at a euchromatic gene, functioning in a developmental context, and provide the first evidence for a non-telomeric function for HOAP.

## Introduction

Eukaryotic genomes are organized into two distinct classes of chromatin [1]. The major class (euchromatin) can undergo decondensation to enable transcription during interphase, whereas a minor fraction (heterochromatin) remains compact and mostly transcriptionally silent throughout the cell cycle. Pericentric and telomeric regions of chromosomes from fungi to humans are organized into a constitutive form of heterochromatin, marked by heterochromatin protein 1 (HP1a in *Drosophila* [2] and humans [3], Swi6 in *S. pombe* [4]) and lysine 9-methylated histone H3 (MeK9H3) [5], [6]. Lysine 9 methylation of histone H3 is catalyzed by the *Drosophila* SU(VAR)3-9 protein [7] (human SUV39H1 [8], *S. pombe* clr4 [9]) and provides a chromatin-binding site for HP1. Both heterochromatin marks have also been observed in euchromatic genes [10], where their roles in gene activation, as well as repression, have recently been uncovered [11]–[13].

The question of how *Drosophila* HP1a (designated HP1 throughout this text) is targeted to specific chromosomal regions prompted our biochemical characterizations of HP1 complexes in the maternally loaded cytoplasm of early embryos [14], [15]. HP1/ORC-Associated Protein (HOAP) was identified as a component of a complex that also contains *Drosophila* origin recognition complex (ORC) subunits [16]. Similarity of the HOAP N-terminus to the HMG-box of mammalian SRY (sex-determining region of the Y chromosome) proteins suggested a role for its DNA-binding activity and that of the ORC, in targeting HP1 to constitutive heterochromatin. Recent data in *Drosophila* and *S. pombe* point to a role for small interfering RNAs (siRNA) from heterochromatin-enriched transposable elements in targeting SU(VAR)3-9 (clr4) and HP1 (Swi6) to these regions [17]–[21]. RNAi-independent mechanisms also operate in recruiting HP1 to heterochromatin and to euchromatic genes [5], [8], [22]–[25]. Indeed, recent data point to a role for the DNA binding activity of KAP-1 (TIF1-β) in targeting HP1 to SRY-regulated genes in repressing transcription of testis-specific genes in the ovary [26].

HOAP is best known for its cooperative role with HP1 in forming a capping complex over *Drosophila* telomeres [27]–[29]. Immunostaining for HOAP also shows the protein at multiple non-telomeric sites in both heterochromatin and euchromatin of larval salivary gland polytene chromosomes [16], [30]. This study was undertaken to examine the non-telomeric functions of HOAP through microarray expression profiling of a mutant for it in order to identify candidate HOAP-regulated genes in these regions [27]. Contrary to our expectation, the majority of genes with altered expression in the mutant had reduced, rather than elevated, transcript levels. The majority of those with reduced transcript levels were found to normally be expressed only in the testis. This led us to uncover an underlying effect of the mutation on male viability and a role for both HOAP and HP1 in regulating the establishment promoter for the master sex determination gene in *Drosophila*, *Sex lethal* (*Sxl*) [31]–[33].

This establishment promoter of *Sxl*, *Sxl_Pe_*, is critical to the sex determination decision which is made early in embryogenesis (see Figure 4A for an overview of the *Sxl* locus). *Sxl_Pe_* is only transcribed in females, which have two X chromosomes. In counting the X chromosome number, also known as the X∶A ratio, *Sxl_Pe_* responds to five X-linked activating genes (*sisterless-a, sisterless-b, runt, myc and unpaired*) working in conjunction with positive maternal factors such as Daughterless. These activating components have their dose measured against the negative effect of maternal factors, such as Groucho and Extramacrochetae, and genes on the autosomes (*deadpan* is the only known member). Firing of *Sxl_Pe_* generates functional SXL protein which initiates the female-mode of splicing of transcripts from the maintenance promoter, *Sxl_Pm_*. *Sxl_Pm_* is transcribed in both sexes, soon after *Sxl_Pe_* shuts down and its mRNAs are being turned over. In female embryos, the SXL protein from *Sxl_Pe_* transcripts inhibits inclusion of the male-specific exon, which would otherwise prematurely terminate translation, of *Sxl_Pm_* transcripts. This autoregulatory splicing loop maintains SXL expression for the rest of the female life cycle. As males do not activate *Sxl_Pe_* they make no SXL protein and the splicing of *Sxl_Pm_* transcripts includes the male exon by default. Through this autoregulation, the binary sex determination decision is maintained.

SXL in females, through splicing and translational regulation, controls the downstream sex determination genes. A vital effect is turning off dosage compensation (DC), which equalizes X chromosome gene dose between the sexes by upregulating transcription of the male X by about two-fold [32], [34]. Failure to activate *Sxl_Pe_* thus leads to the improper, male mode of splicing of *Sxl_Pm_* transcripts and female lethality. Conversely, inappropriate activation of it in males results in the female mode of splicing of *Sxl_Pm_* transcripts and male lethality.

Our data show roles for both HOAP and HP1 in regulating the critical decision of whether activation of *Sxl_Pe_* will occur. These data support HOAP acting as a repressor, and HP1 as an activator at this promoter. They also suggest that HP1 first cooperates with HOAP in repressing *Sxl_Pe_* before switching to an activation mode. This is the first report of a non-telomeric function for HOAP and the most precisely defined role to date for HP1 in developmental control of a euchromatic gene.

## Results

### Decrease in Testis-Specific Transcripts Reflects Under-Representation of *cav* ^−^ Males

In an effort to identify candidate HOAP-regulated genes, the Affymetrix *Drosophila* Genome Array 1 was used to compare the expression profile of wild type larvae to those that were mutant for the HOAP-encoding gene (*caravaggio* or *cav*). The original recessive lethal *cav* ^1^ allele was used in the study. This allele contains a 5 bp insertion that causes truncation of the HOAP protein after two of three copies of a C-terminal HP1-binding repeat [27], [30]. The *y* ^1^ *w* ^67c23^ stock, which provides the genetic background for all genetic manipulations in the lab, was used as the wild type control. Larvae of each genotype were collected at the first and second instar stage, prior to the lethal phase of the *cav* ^1^ mutant. Out of 13,500 transcription units represented on the array, 183 genes were found to have significantly altered expression (log_2_R+2.0, p<0.01) in *cav^1^* mutant larvae. Within this set, 142 genes had reduced transcript levels and 41 had increased levels.

Two strategies were then used to catalogue the normal expression profiles of genes in each data set. A gene's relative representation in publically available tissue-specific cDNA libraries [35]–[37] provided the first method for assessing its normal tissue distribution. This analysis was later complemented by data from two published microarray profiling studies of *Drosophila* sex- or tissue-specific gene expression [38], [39]. The results of these analyses are summarized in Tables S1 and S2. Genes categorized as “multiple” were represented in cDNA libraries of at least three different developmental specificities and enriched in at least three different tissue types in the Chintapalli et al. study [38]. Those categorized as “rare” were represented by a single or few cDNA clones and detected at low levels in multiple tissues by Chintapalli et al. [38]. Those categorized as tissue-specific (e.g., testis or midgut) were represented only, or predominantly, in cDNA libraries of that tissue-specificity and also specifically enriched in that tissue in Chintapalli et al. [38]. The most striking pattern to emerge from these analyses was the relative enrichment of testis-enriched genes (67%) in gene set with reduced transcript levels in the *cav^1^* mutant. The Parisi et al. [39] study of sex-specific gonad expression provided corroboration for the testis-specificity of 67% of these genes, as they were both enriched in testes relative to whole animals and in testes relative to ovaries. This is in contrast to a complete absence of testis-specific genes in the gene set with elevated transcript levels, and also far exceeds the ∼12% of *Drosophila* genes reported to have testis-specific expression [40].

The enrichment of testis-specific genes in the reduced transcript level data set could indicate a requirement for HOAP in testis-specific gene expression. Alternatively, it could reflect an early lethal phase for *cav* ^1^ mutant males and, thus, under-representation of male-specific transcripts in the *cav* ^1^ RNA sample. RNA interference (RNAi) against the HP1-encoding gene (*Su(var)205*) in *Drosophila* has revealed enhanced vulnerability of males to partial HP1 knockdown [41]. To determine if males were under-represented in our *cav^1^* larval sample, we used the X-linked *yellow* (*y* ^+^) marker to sex individual *cav* ^1^ mutant larvae, allowing us to sex the animals through a phenotype other than gonad size. By this criterion, 2.83-fold fewer male *cav* ^1^ larvae were observed than female *cav* ^1^ larvae. Using PCR with Y-linked primers to sex individual *cav* ^1^ homozygous embryos, we also observed approximately two-fold more male embryos fail to progress to the larval stage.

**Figure 1:**
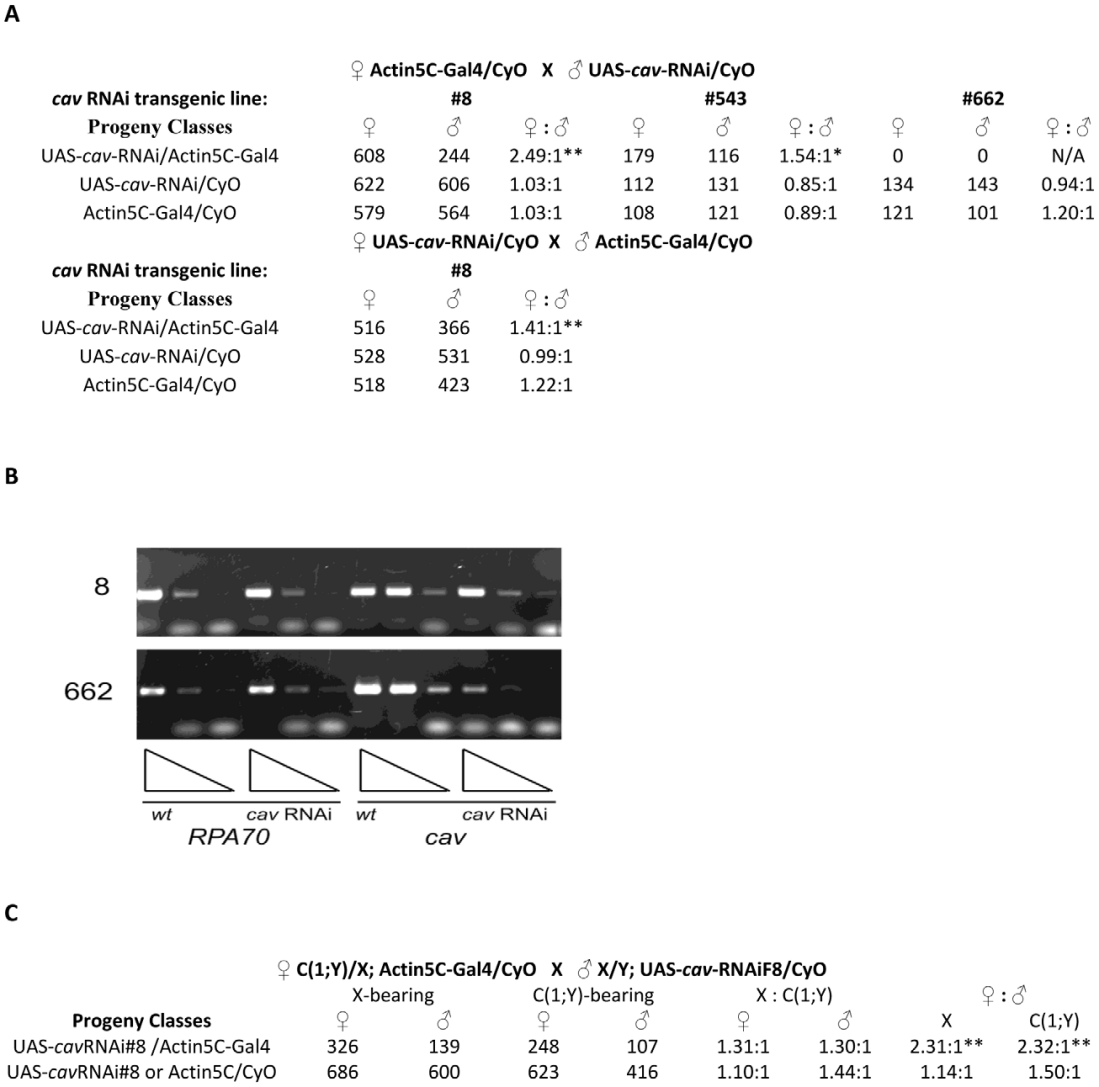
*cav* RNAi reduces adult male viability. (A) The GAL4/UAS binary system using maternally contributed Actin-5C GAL4 to drive expression of *cav* RNAi shows reduced viability of males carrying the GAL4 driver and *cav* RNAi transgene #8; both sexes carrying the GAL4 driver and *cav* RNAi transgene #662 had reduced viability, in comparison to comparison to their control siblings containing either *cav* RNAi or Act5C-GAL4 transgene alone. (p<0.01^**^) Male viability was also significantly reduced (albeit less dramatically) in the reciprocal cross in which the Actin-5C GAL4 transgene was paternally derived. (p<0.01^**^) (B) RT-PCR assays of endogenous *cav* and *RpA70* (normalizing standard) in larvae expressing *cav* RNAi transgenes from lines #8 and #662. The triangles represent a 5-fold dilution series of RNA from animals carrying either the *cav* RNAi transgene only (wt) or *cav* RNAi and Act5C-GAL4 (*cav* RNAi). (C) Effect of the compound X-Y chromosome, C(1;Y), in animals expressing *cav* RNAi. The presence of C(1;Y) did not significantly affect viability of adult females or males expressing *cav* RNAi, in comparison to their control siblings containing either *cav* RNAi or Act5C-GAL4 transgene alone (p>0.10). By contrast, the animal's sex significantly affected viability, only in the class of animals expressing *cav* RNAi, regardless of whether they did or did not carry C(1;Y). (p<0.01^**^).

We then used RNAi to partially knockdown HOAP and determine its effect on adult male viability. The GAL4/UAS binary expression system was used to drive expression of a *cav* RNAi transgene (Figure 1). Ubiquitous expression of *cav* RNAi from two transgenic lines (F8 and 543) through a maternally introduced Actin5C GAL4 driver resulted in a 2.5- and 1.5-fold reduction in adult male viability relative to females, respectively (Figure 1A). A third *cav* RNAi transgenic line (662) resulted in lethality of both males and females. The severity in effect of *cav* RNAi expression in different transgenic lines correlated with the degree to which endogenous *cav* mRNA was reduced as monitored through semi-quantitative RT-PCR, with ∼80% *cav* knockdown resulting in a 2.5-fold reduction in male viability (Figure 1B). The dose-dependent effects of *cav* RNAi expression on male viability are similar to those observed with *Su(var)205* RNAi expression (2.54-fold reduction with 60–90% *Su(var)205* knockdown and lethality of both sexes with >90% knockdown) [41]. A less severe, but significant, reduction in viability of male progeny was also observed in the reciprocal cross in which the Actin5C GAL4 transgene was introduced through the fathers. Although the results in both sets of crosses indicate a zygotic requirement for HOAP in male viability, the more pronounced effect was with maternally contributed Actin5C GAL4. This suggests maternally expressed GAL4 is needed to drive earlier expression of the *cav* RNAi for maximum effect.

A dominant effect on male viability was also observed for a newly recovered *cav* allele (*cav* ^2248^). Heterozygosity for this allele reduces male viability 1.84-fold (n≥312, p<0.01). The *cav* ^2248^ carries a nonsense mutation at nucleotide 111 of the *cav*-PB coding sequence within the region of similarity to the SRY HMG box. This allele appears to exert dominant negative activity on the wild type protein, as its effect on male viability is similar to that caused by RNAi-induced HOAP knockdown but is more pronounced than a deficiency for the locus (Df(3R)F89-4). The prematurely truncated protein in this mutant, or a reinitiation product from the next Met within the HMG box, is apparently responsible for this dominant negative effect. Smaller, but significant, reductions in male viability were also observed in progeny from wild type crossed to either parent carrying the *cav* ^2248^ allele (data not shown). The lethal phase for *cav* ^2248^ heterozygous males was determined to be late in embryogenesis after denticle belt formation; *cav* ^2248^ homozygous embryos had an earlier lethal phase which is apparently due to telomeric defects (Figure S1).

### Presence of Y Chromosome Is Not Responsible for Reduced Male Viability

Although the Y chromosome is not required for viability or sexual differentiation in *Drosophila* males, its heterochromatic composition enables it to act as a sink for heterochromatin proteins in suppressing position effect variegation of euchromatic genes artificially juxtaposed to heterochromatin [42], [43]. A limited pool of heterochromatin proteins might then render males more vulnerable to reductions in HOAP (and other heterochromatin proteins). To test whether the Y chromosome has a role in reducing viability of males that are deficient for HOAP, a compound X-Y chromosome [C(1;Y)] was introduced into animals expressing a *cav* RNAi transgene (Figure 1C). While the C(1;Y) chromosome modestly reduced viability of both male and female progeny by 30% relative to siblings lacking it, the sex of the animal had a pronounced effect on viability. Males expressing *cav* RNAi showed a 2.3-fold decrease in viability, regardless of whether they also carried C(1;Y).

### Defects in Sex Determination Pathway Are Responsible for Reduced Male Viability

Defects in the sex determination pathway, causing inappropriate dosage compensation in *Drosophila*, result in sex-specific lethality. Sex-lethal (SXL) protein acts as the master switch regulator of this pathway at the level of splicing and translation [44]. As described earlier, the critical decision is made early in embryogenesis by the activation of the *Sxl* establishment promoter (*Sxl_Pe_*) in only females, to generate functional SXL protein [32]. Failure to activate *Sxl_Pe_*, thus leads to improper male splicing of *Sxl_Pm_* transcripts and female lethality. Conversely, inappropriate activation of *Sxl_Pe_* in males results in the female mode of splicing of *Sxl_Pm_* transcripts and male lethality.

**Figure 2:**
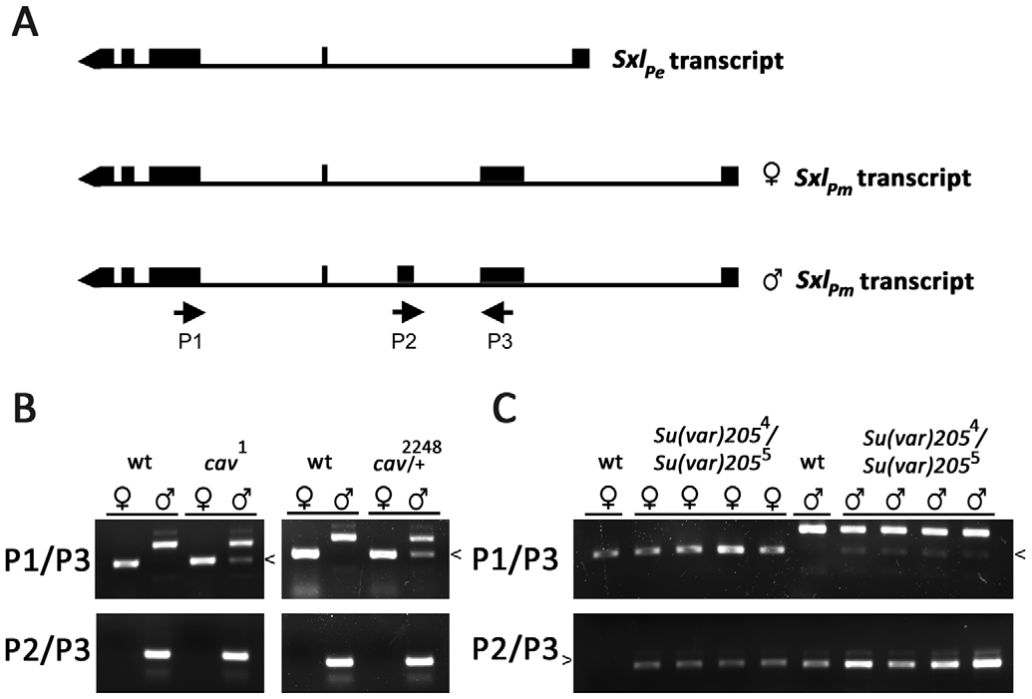
Aberrant sex-specific *Sxl_Pm_*-derived transcripts are observed in mutants for HOAP and in mutants for HP1. (A) RT-PCR was used to specifically amplify male- or female-specific *Sxl_Pm_* transcripts. The P1/P3 primer pair amplifies a 482 bp product from the female-specific transcript and a 672 bp product from the male-specific transcript in wild type animals (labeled wt in all three panels). The P2/P3 pair amplifies a 284 bp product from a male-specific transcript in males only. (B) Pools of RNA from individually sexed male or female *cav* ^1^ homozygous or *cav* ^2248^ heterozygous embryos failing to progress to the larval stage were used in RT-PCR assays with both P1/P3 (top panel) and P2/P3 (bottom panel) primer pairs, revealing the presence of aberrant female-specific *Sxl_Pm_* transcripts in male embryos (arrowhead). RT-PCR assays of RNA from four different individually sexed *Su(var)205* ^4^/*Su(var)205* ^5^ female (♀) or male (♂) larvae, each with both P1/P3 (top panel) and P2/P3 (bottom panel) primer pairs revealed the presence of aberrant sex-specific *Sxl_Pm_* transcripts in both sexes. The methods used to genotype and sex individual embryos and larvae are described in Materials and Methods.

To determine if the enhanced male lethality of *cav* mutants is associated with inappropriate SXL activity in males, RT (reverse transcriptase)-PCR assays were used to monitor sex-specific splicing of *Sxl_Pm_* transcripts in heterozygous *cav* ^2248^ and homozygous *cav* ^1^ male embryos that failed to progress to the larval stage. Individual embryos of the appropriate genotype were identified through a GFP-marked wild type balancer chromosome (described more fully in Materials and Methods) and sexed by PCR with Y-linked primers (Figure S2). RNA was then purified from pools of male or female embryos and used in RT-PCR assays with *Sxl* primer pairs (P1/P3 and P2/P3) designed to discriminate between *Sxl_Pm_* transcripts spliced in the male vs. female mode (Figure 2A). Assays of RNA from wild type (*y* ^1^ *w* ^67c23^) embryos showed correct sex-specific splicing of *Sxl_Pm_* transcripts in both males and females (Figure 2B). By contrast, a minor fraction of transcripts had undergone the female-mode of splicing in heterozygous *cav* ^2248^ and homozygous *cav* ^1^ male embryos (arrowhead in Figure 2B).

If inappropriate SXL expression is the cause of the enhanced male lethality in the *cav* ^2248^ heterozygous males, we would expect a loss of function *Sxl* allele to rescue them. To this end, the *Sxl* ^f1^ point null allele was introduced into heterozygous male and female *cav* ^2248^ progeny through their mothers. Closely linked recessive markers, *cut* and *carmine*, which flank *Sxl* were used to follow the *Sxl* ^f1^ allele in males, while in females *white^+^*, which should segregate with *Sxl* ^f1^ 82.3% of the time, was used as a close approximation. As shown in Table 1, the viability of *cav* ^2248^ males carrying a defective *Sxl* allele (*Sxl* ^f1^) was not significantly different from their female siblings, unlike their *Sxl^+^* brothers (*yw*). It should be noted that in this cross, the effect of the *cav* ^2248^ mutation on male viability was somewhat reduced; we ascribe this difference to the temperature at which the cross was done. For reasons that are not clear at present, all progeny from this cross were nonviable at 25°C, so the cross was performed at room temperature. An overall rescue in male viability was also observed by the introduction of the *Sxl* ^fP7BO^ allele, although for this cross the markers on the deletion allele chromosome did not allow identification of the different progeny classes.

**Table 1:** Mutations in HOAP reduce male viability, and loss of *Sxl* partially rescues the lethality.

♀ *yw*; *cav* ^2248^/TM3 X ♂ *yw*; *cav* ^2248^/TM3		
♀	♂	♀ ∶ ♂
202	110	1.84 ∶ 1**

♀ *Sxl* ^−^/*yw*; *cav* ^2248^/TM3 X ♂ *yw*/Y; *cav* ^2248^/TM3					
♀		♂		♀ ∶ ♂	
*Sxl* ^f1^	*yw*	*Sxl* ^f1^	*yw*	*Sxl* ^f1^	*yw*
317	311	276	232	1.15 ∶ 1	1.34 ∶ 1*

The ratio of male to female *cav* ^2248^ heterozygous progeny differs significantly from the expected ratio; introduction of the *Sxl* ^f1^ allele to these progeny restored the expected ratio. (p<0.05^**^, <0.10^*^).

As HP1 and HOAP interact, and HP1 reduction also preferentially affects male viability, we wondered whether male larvae mutant for the HP1-coding gene [*Su(var)205* ^5^/*Su(var)205* ^4^] would also show altered splicing of *Sxl*_Pm_ transcripts. GFP-marked balancer chromosomes were used to identify individual larvae of the correct genotype, and both an X-linked genetic marker (*y^+^*) and PCR assays of a Y-linked gene were used to sex them. Surprisingly, we found aberrant *Sxl*_Pm_ transcripts in multiple individual *Su(var)205* ^5^/*Su(var)205* ^4^ larvae of both sexes (arrowheads in Figure 2C), suggesting HP1 has a dual role, of opposite consequence in each sex.

We therefore explored whether a maternal mutation in either *cav* or *Su(var)205* would affect the viability of female progeny that also have a reduction in *Sxl* dose (Table 2). Female viability is not compromised in a cross between either wild type females and *Sxl^−^* males (fP7B0 or f1), or between *cav* mutant females and *Sxl* ^−^/Y fathers (Note: the modest reduction in male viability in the progeny from *cav* ^2248^ females and *Sxl^−^* males is essentially the same as that observed in progeny from *cav* ^2248^ females and wild type males, as described earlier). However, in progeny from *Su(var)205* heterozygous females crossed to *Sxl^−^*/Y males, female viability was dramatically reduced, particularly with the strong loss of function *Su(var)205* ^5^ allele. The effect of *Su(var)205* mutations is strictly maternal; no significant reduction in female viability is observed in the reciprocal cross (Table 2). Interestingly, this effect is allele-specific. Whereas mothers carrying the *Su(var)205* ^5^ null allele or the *Su(var)205* ^4^ carboxyl-terminally deleted allele had greatly reduced viability in their female progeny, mothers carrying a point mutation in the MeK9-H3-binding chromodomain allele [*Su(var)205* ^2^] had a modest, but significant, effect. Although neither *cav* allele affected sex-specific viability of progeny from *Sxl* mutant fathers, the introduction of either allele into *Su(var)205* mothers rescued the *Su(var)205* maternal effect on female viability (Table 3). Both the dominant negative *cav* ^2248^ allele and the C-terminally truncated *cav* ^1^ allele, with compromised HP1-binding activity [27], [30], were capable of rescuing the *Su(var)205* maternal effect, and this rescue was also strictly maternal.

**Table 2:** Mutations in HP1 interact with loss of function *Sxl* alleles to reduce female viability.

♀ *Su(var)205^−^*/CyO X ♂ *Sxl* ^fP7B0^/Y						
Progeny:	♀		♂		♀ ∶ ♂	
maternal allele	*Su(var)205*	CyO	*Su(var)205*	CyO	*Su(var)205*	CyO
*Su(var)205^5^*	14	31	231	230	1 ∶ 16.5**	1 ∶ 7.42**
*Su(var)205* ^4^	82	88	325	281	1 ∶ 3.96**	1 ∶ 3.19**
*Su(var)205* ^2^	154	152	224	192	1 ∶ 1.45*	1 ∶ 1.26

♀ *Su(var)205^−^*/CyO X ♂ *Sxl* ^f1^/Y						
Progeny:	♀		♂		♀ ∶ ♂	
maternal allele	*Su(var)205*	CyO	*Su(var)205*	CyO	*Su(var)205*	CyO
*Su(var)205^5^*	2	9	122	120	1 ∶ 61.0**	1 ∶ 13.3**
*Su(var)205* ^4^	143	141	233	210	1 ∶ 1.63**	1 ∶ 1.49**

♀ *Sxl* ^fP7B0^/FM7 X ♂ *Su(var)205* ^−^/CyO						
Progeny:	♀		♂		♀ ∶ ♂	
paternal allele	*Su(var)205*	CyO	*Su(var)205*	CyO	*Su(var)205*	CyO
*Su(var)205^5^*	93	85	120	105	1 ∶ 1.29	1 ∶ 1.24

Female viability is markedly reduced in progeny from *Su(var)205* mothers and *Sxl* fathers (Maternal) but not in progeny from *Su(var)205* mothers and *Sxl^−^* fathers (Paternal). (p<0.05^**^, <0.10^*^). For the third cross, numbers of FM7 classes not presented as the balancer also affects viability (males in particular) and these progeny were not factored in the female to male comparison.

**Table 3:** Maternal mutations in HOAP and HP1 have antagonistic effects on female viability.

♀ *Su(var)205^5^*/CyO; *cav* ^− ^X ♂ *Sxl* ^fP7B0^/Y			
maternal allele	♀	♂	♀ ∶ ♂
*cav* ^2248^	231	200	1.15 ∶ 1
*cav* ^1^	42	172	1 ∶ 4.09

♀ *Su(var)205^5^*/CyO X ♂ *Sxl* ^fP7B0^/Y; *cav* ^−^			
maternal allele	♀	♂	♀ ∶ ♂
*cav* ^2248^	8	187	1 ∶ 23.0**
*cav* ^1^	6	202	1 ∶ 34.0**

Maternal (Maternal), but not paternal (Paternal), introduction of a *cav* mutant allele to *Su(var)205* mothers rescues viability of female progeny from *Sxl^−^* fathers. (p<0.01^**^).

### Transcription of *Sxl_Pe_* Is Affected by Mutations in Either *cav* or *Su(var)205*

**Figure 3:**
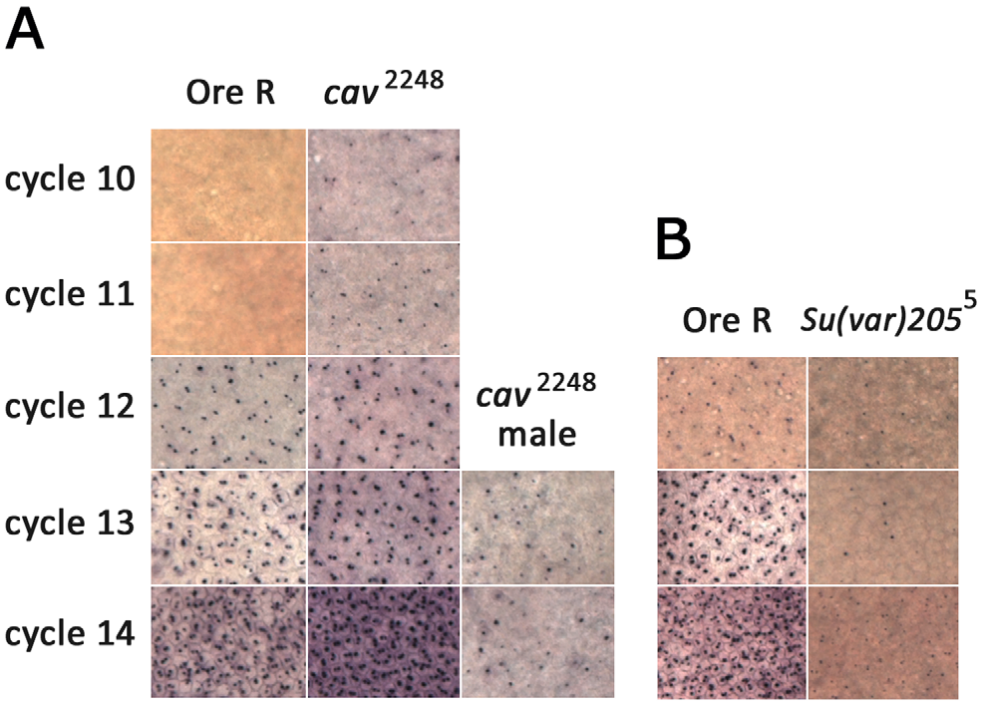
*In situs* for *Sxl_Pe_* transcripts in embryos from wild type, *cav^2248^*/TM3, Sb, or *Su(var)205* ^5^/CyO parents. Comparisons of the same-sized area of images taken at 40×. (A) *Sxl_Pe_* transcripts are present only in Ore R wild type females (containing 2 dots per nucleus) during cycle 12 to 14. *Sxl*_Pe_ transcripts are present in female embryos from *cav^2248^*/TM3 parents as early as cycle 10. *Sxl_Pe_* transcripts are also frequently present in male embryos from *cav^2248^*/TM3 parents (single dot per nucleus, usually near nuclear periphery where the dosage compensated X chromosome resides [62]. Not much signal is detected before cycle 13 in males. (B) Poor *Sxl_Pe_* expression is observed in embryos from *Su(var)205* ^5^/CyO parents in comparison to simultaneously stained embryos from Ore R wild type parents.

The rescue of *cav* ^2248^ male viability by the loss of *Sxl* and the antagonistic maternal effects of *cav* and *Su(var)205* on the viability of their *Sxl* ^−^-bearing female progeny, suggested a role for maternal HOAP and HP1 in regulating *Sxl_Pe_*. To directly assess the effect of reduced HOAP or HP1 on *Sxl_Pe_*, *in situ* hybridizations were performed with probes that distinguish the early from maintenance *Sxl* mRNAs on 0–4 hr embryos from *cav* ^2248^ and *Su(var)205* ^5^ heterozygous parents. For *Sxl_Pe_*, wild type embryos show two dots on the chromosomes, one for each female X, beginning in cycle 12 through to early cycle 14. Cycle 14 is also when the maintenance promoter, *Sxl_Pm_*, is activated and the autoregulatory splicing loop set in motion in females. In embryos from *cav* ^2248^ heterozygous parents, two key changes were observed (Figure 3A). First, expression of *Sxl_Pe_* in females began two cycles earlier than normal – cycle 10, and overall expression appeared more robust than in wild type embryos. Second, male embryos also showed *Sxl_Pe_* activation (single *in situ* dot), although the expression was not as strong or as early as in females. The level of expression in male embryos was more variable both between embryos and at the level of individual nuclei. The majority had sporadic or scattered positive nuclei, while others had much greater numbers, as shown in Figure 3A. A count of early cycle 14 male embryos suggests >95% of the male embryos (n = 33) had at least some positive nuclei. In embryos from *Su(var)205* ^5^ heterozygous parents, *Sxl_Pe_* expression was not observed in male embryos (single *in situ* signal) and was weaker and more variable than normal in females (Figure 3B). Analysis of the female embryos indicates that ∼85% express the promoter weakly during cycles 12 and 13 (n = 14–20 for each cycle) and less than half the embryos have normal levels of expression at cycle 14 (n>25). These results are consistent with the early expression of *Sxl_Pe_* being heavily reliant on the maternal deposit of HP1 protein and/or mRNA, with the zygotic contribution becoming more apparent at cycle 14. Expression of the maintenance *Sxl_Pm_* transcripts did not show significant changes in embryos from either genotype (Figure S3). Although HP1 binds to *Sxl_Pm_* in 4–18 hr embryos, at cellular blastoderm the promoter does not appear to be sensitive to a reduction in maternal HP1.

### Both HOAP and HP1 Are Associated with the *Sxl*_Pe_ Regulatory Region

**Figure 4:**
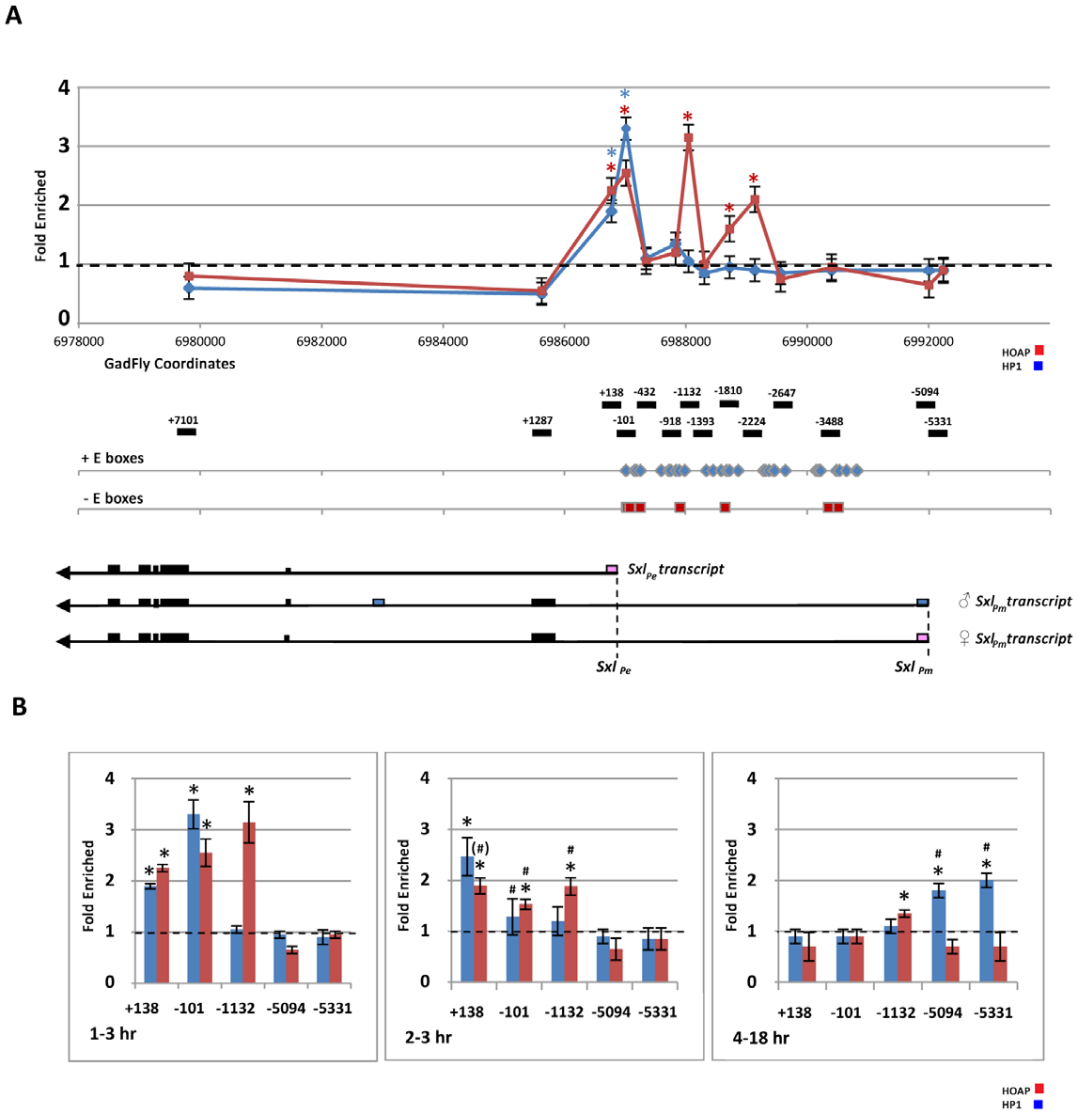
Chromatin immunoprecipitation (ChIP) assays show association of both HOAP and HP1 with the *Sxl* locus. (A) Relative enrichment (ChIP/input-*Sxl* sequence/ChIP/input-RpA-70) of specific *Sxl* locus sequences in HOAP- and HP1-ChIP fractions from 1–3 hr embryos plotted against the molecular map of the *Sxl* locus (HOAP in red; HP1 in blue). The positions of *Sxl* fragments monitored for enrichment are shown relative to the *Sxl_Pe_* initiation site, below the map. The positions of E-box binding sites for positive (blue diamonds) and negative (red squares) bHLH factors [63] and *Sxl* transcripts are also indicated below the map. (p_(no enrichment)_<0.05*) (B) Bar graphs show enrichment of *Sxl* sequences in HOAP- (red bars) and HP1- (blue bars) ChIP fractions during development (1–3 hr, 2–3 hr, and 4–18 hr staged embryos). (p_(no enrichment)_<0.05*; change between 1–3 hr and 2–3 hr embryos, p_(no effect)_ p_(no change)_<0.10^(#)^, <0.05^#^).

Chromatin immunoprecipitation assays were then used to determine if the effects of *cav* ^2248^ and *Su(var)205* ^5^ on *Sxl_Pe_* activity are mediated through direct physical association of the proteins with the *Sxl* locus (Figure 4). Cross-linked chromatin was prepared from developmentally staged collections of wild type (*y* ^1^ *w* ^67c23^) embryos and immunoprecipitated with antibodies against HP1, HOAP, and non-immune IgG. Embryos were staged to monitor both *Sxl* promoters: before and during *Sxl_Pe_* activation (1–3 hr), during *Sxl_Pe_* activation (2–3 hr), and during *Sxl_Pm_* expression (4–18 hr). Quantitative real time PCR was then used to measure enrichment of *Sxl* sequences in each ChIP fraction. As summarized in Figure 4A, significant enrichment of sequences in the immediate vicinity of *Sxl_Pe_* (+138 and −101 fragments) was observed in the HP1 and HOAP ChIP fractions but not in the IgG ChIP fraction from 1–3 hr embryos. No enrichment of *Sxl_Pm_* sequences was observed at this stage. Significant enrichment of sequences upstream of *Sxl_Pe_* (−1132,−1800, and −2224) was also observed in the HOAP ChIP fraction. Using an anti-Myc antibody to probe chromatin from a stock expressing Myc-tagged HOAP from the endogenous *cav* locus [29], similar results were obtained (data not shown).

A comparison of the ChIP data from 1–3 hr versus 2–3 hr embryos shows a significant decrease (p<0.5) in the associations of both HOAP and HP1 with the −101 region of *Sxl_Pe_*_._ Both proteins retained significant associations with the +138 region of *Sxl_Pe_* in 2–3 hr embryos at a time that is coincident with *Sxl_Pe_* expression. HOAP enrichment in both the +138 region and the −1132 region appeared reduced at this time, although only at the 90% confidence level. No enrichment of either protein was found more 3′ in *Sxl* gene coding sequences or in the *Sxl_Pm_* region in either the 1–3 hr or 2–3 hr embryos. Also, *Sxl_Pe_* sequences were not enriched in either the HP1 or HOAP ChIP fractions from 4–18 hr embryos. However, HP1 enrichment in the vicinity of *Sxl_Pm_* (−5094 and −5331) and a slight, but significant, enrichment of HOAP in the −1132 region was observed in later staged embryos.

### HOAP and HP1 Affect *Sxl_Pe_* Chromatin

The ChIP assays described above confirmed physical association of HOAP and HP1 with the *Sxl_Pe_* promoter, but do not distinguish between the sexes. We, therefore, took advantage of the feminizing and masculinizing effects of maternal mutations in *cav* and *Su(var)205* to gain insight into the requirement for each protein in regulating *Sxl_Pe_* activity.

**Figure 5:**
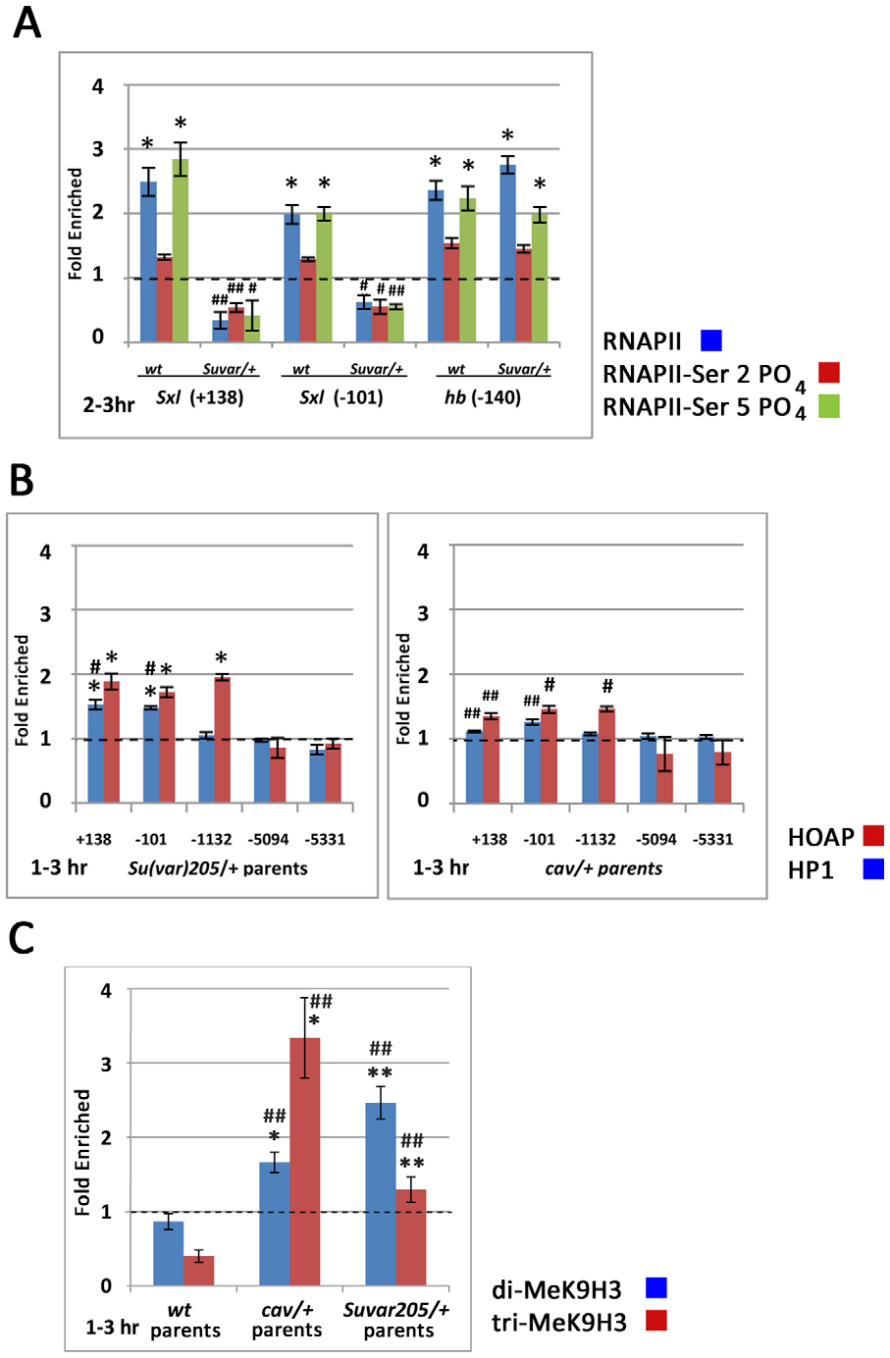
Chromatin immunoprecipitation (ChIP) assays show perturbations in *Sxl*_Pe_ chromatin in embryos from HOAP and HP1 mutant mothers. (A) Bar graphs show *Sxl_Pe_* (*Sxl* +138 and −101) and *hunchback* (*hb* −140) promoter sequences in RNA polymerase II ChIP fractions from embryos produced by either wild type (wt) or *Su(var)205* ^5^/+ mothers crossed to *Sxl* ^f1^/Y fathers. ChIP assays for unphosphorylated (blue bar), Ser2-phosphorylated (red bar), or Ser5-phosphorylated (green bar) RNA polymerase II are shown. (p_(no enrichment)_<0.05*; change between wild type and mutant embryos, p_(no change)_<0.05^#^, <0.01^##^) (B) Bar graphs show changes in the enrichment of *Sxl* sequences in HOAP- (red bars) and HP1- (blue bars) ChIP fractions in embryos produced by *Su(var)205* ^5^/CyO parents (left) and by *cav* ^2248^/TM3, Sb parents (right). (p_(no enrichment)_<0.05*; change between wild type and mutant embryos, p_(no change)_<0.05^#^, <0.01^##^) (C) Bar graphs show changes in the enrichment of *Sxl* sequences in di-MeK9H3- (blue bars) and tri-MeK9H3- (red bars) ChIP fractions in embryos produced by wt, *Su(var)205* ^5^/CyO and *cav* ^2248^/TM3, Sb parents. (p_(no enrichment)_<0.05*; change between wild type and mutant embryos, p_(no change)_<0.05^#^, <0.01^##^).

The *in situs*, as well as the lethal effect of a maternal mutation in *Su(var)205* on female progeny lacking one functional copy of *Sxl*, indicated an activation function for HP1 at *Sxl_Pe_*. To further test this hypothesis and examine its nature, we determined the effect of reducing maternal HP1 on RNA polymerase II (RNAP II) association with *Sxl_Pe_* (Figure 5A). Chromatin was prepared from 2–3 hr. embryos from wild type or heterozygous *Su(var)205* ^5^ mothers mated to *Sxl* ^f1^ fathers, as *Sxl_Pe_* is active at this stage in wild type embryos. Antibodies that recognize three different phosphoisoforms of RNAP II, which mark distinct stages of transcription initiation and elongation, were used to gain insight into the level of transcriptional activation at *Sxl_Pe_* at which HP1 is functioning. In embryos from wild type mothers, both unphosphorylated RNAP II (blue bar) and Ser 5-phorphorylated carboxyterminal domain RNAP II (green bar), which marks early steps of transcription elongation, were enriched in the proximal regions of *Sxl_Pe_* and the *hunchback* (*hb*) promoter. Both genes are active at this stage. Ser 2-phosphorylated RNAP II (red bar) which identifies polymerase in its elongation phase was mostly absent from both promoters. All RNAP II isoforms, particularly those associated with transcription initiation (unphosphorylated RNAP II, blue bars) and early events in transcription elongation (Ser 5-phosphorylated RNAP II, green bars), were drastically reduced from *Sxl_Pe_* proximal sequences in embryos from *Su(var)205* mutant mothers and *Sxl* ^f1^ */Y* fathers (Figure 5A). By contrast, the *Su(var)205* mutation did not affect RNA polymerase II association with the *hb* promoter (Figure 5A).

The feminizing effects of reducing maternal HOAP, most notably the effect of the *cav* ^2248^ mutation on transcription from *Sxl_Pe_*, support a repressive role for HOAP at this promoter. The partial rescue of the maternal *Su(var)205* masculinizing effect by the HP1-binding-defective *cav* ^1^ allele implicates HP1 in this repression as well. To examine the interdependency of HOAP and HP1 in their association with *Sxl_Pe_* sequences, we performed ChIP assays of each protein in 1–3 hr embryos with a reduced maternal dose of the other protein. As shown in Figure 5B, a maternal mutation in one copy of the gene for either protein significantly reduced association of its encoded protein with *Sxl_Pe_* sequences. These data also indicate a strong reliance of HP1 on HOAP for its association with *Sxl*_Pe_ proximal sequences in embryos of this stage. HP1 association with these sequences was significantly reduced in embryos from *cav* ^2248^ heterozygous parents. HOAP association with *Sxl_Pe_* sequences, also appeared educed in embryos from *Su(var)205* ^5^ heterozygous parents, but this reduction was not statistically significant. These data support a role for HOAP in recruiting HP1 to *Sxl_Pe_* prior to the time of *Sxl_Pe_* activation; failure to form this repressive chromatin in embryos from heterozygous *cav* ^2248^ mothers apparently negates the need for HP1 in *Sxl*_Pe_ activation slightly later in embryonic development.

K9-methylated histone H3 has a well known conserved role in HP1 association with constitutive pericentric heterochromatin [7]–[9] and some euchromatic genes [5]. The activating function of HP1 at *Sxl*_Pe_ does not appear to rely on this histone modification, as a maternal mutation for the *Su(var)205* ^2^ allele, which encodes a protein that is defective for MeK9H3-binding, does not strongly affect the viability of female progeny from *Sxl* mutant fathers. It is still possible, however, that HP1-binding to MeK9H3 has a role in HP1 repression prior to the time of *Sxl*_Pe_ activation. We, therefore, used ChIP assays to monitor the presence of both di- and tri-methylated histone H3 at the major site of HP1enrichment in the −101 region of *Sxl_Pe_*. The *hb* promoter, which is active at the same time as *Sxl_Pe_* but does not appear to require HP1 for activation, was used as the normalizing standard in these experiments.

Di-MeK9H3 was detected at above background levels (p<0.05) at *Sxl_Pe_*, but not at the simultaneously expressed *hb* promoter, in embryos from wild type mothers (Figure 5C). Although no significant enrichment was observed at *Sxl_Pe_* relative to *hb* in wild type embryos, a maternal *cav* ^2248^ or *Su(var)205* ^5^ mutation resulted in significant enrichment of both di- and tri-MeK9H3 at *Sxl_Pe_* (Figure 5C). Assuming, as in pericentric heterochromatin, a repressive activity for these histone modifications at *Sxl_Pe_*, their increased enrichment in this region in mutants for HP1 and HOAP may reflect a feedback mechanism to compensate the loss of HP1.

## Discussion

The canonical heterochromatin protein HP1 is most commonly associated with constitutive heterochromatin and gene repression. Here we report a critical role for it in regulating one of the earliest decisions in metazoan development, whether to embark on a female or male path of sexual differentiation and dosage compensation. The role of heterochromatin in mammalian dosage compensation has been recognized since the early work of Lyon [45]. Although *Drosophila* utilizes a different mechanism to equalize X-linked gene dose, through hyper-activation of the single male X chromosome via chromatin modification [46], this study provides the first evidence of a role for heterochromatin proteins in the early events of *Drosophila* sex determination. HP1, together with its telomere partner HOAP, influence the critical decision in sex determination - activation of *Sxl_Pe_*, the *Sxl* establishment promoter.

### Heterochromatin Proteins in Sex-Specific Viability

We find that reductions in HOAP preferentially compromise male viability. This was observed for two different *cav* mutant alleles and by reducing HOAP through RNAi. The presence of *Sxl_Pm_*-derived transcripts that have been spliced in the female mode in *cav* mutant males suggested inappropriate *Sxl* activation to be responsible for this reduced viability. In situ data indicating inappropriate firing of *Sxl_Pe_* in male embryos from *cav* ^2248^ heterozygous parents support this view, as does the rescue of the *cav* ^2248^ male viability defect by *Sxl* loss of function mutations. The more pronounced male lethality observed from reducing HOAP by RNAi expression driven by maternal, versus paternal, contribution of Actin5C GAL4 is consistent with such an early requirement for HOAP for male viability (Figure 1).

Previous reports have shown that reducing HP1 by RNAi similarly reduces male viability preferentially [41]. RT-PCR assays of *Sxl_Pm_* transcripts in HP1 mutants, however, suggested a more complex scenario as incorrect sex specific transcripts were observed in both sexes (Figure 2C). This pointed to an activation, as well as repressor, role for HP1. Consistent with an activation role, reduction of maternal HP1 severely compromised female viability when the dose of *Sxl* was also reduced in the progeny (Table 2), and ChIP assays of embryos from this cross showed recruitment of RNAP II to *Sxl_Pe_* to be impaired (Figure 5A). This effect of reducing HP1 on female viability was strictly maternal, as was the antagonizing effect of simultaneously reducing maternal HOAP (Table 3). Moreover, the partial rescue of the *Su(var)205* maternal effect by the C-terminally truncated *cav* ^1^ allele, which produces a protein that is compromised for HP1-binding [27], [30], points to an involvement of HP1 in the antagonizing activity of HOAP. Finally, ChIP assays show a dependence of HP1 on HOAP for its association with *Sxl_Pe_*. Combined, these data indicate both antagonistic and cooperative roles for these heterochromatin proteins in regulating *Sxl_Pe_*, whereby HOAP acts as a repressor and HP1 acts as both an activator and repressor. The reliance of HP1 on HOAP for recruitment to the promoter would suggest HOAP may also have a role in the activation function of HP1 at the promoter, although this was not readily apparent in our assays.

Although our data clearly show maternal roles for HOAP and HP1 in regulating the activity of *Sxl_Pe_*, for both HOAP and HP1 [41] the RNAi knockdown data indicate a substantial zygotic component in their effects on male viability. These zygotic effects, observed only in progeny carrying both an interference RNA transgene and a GAL4 driver transgene (Figure 1A), suggest additional later sex-specific roles for both proteins. Such roles could be related to those observed for HP1 and SU(VAR)3-7 in male dosage compensation [47]. Because the effect of reducing these proteins on the chromosomal distribution of DCC proteins [47] is the opposite of those observed for males that are deficient for DCC proteins [48], as predicted to occur with inappropriate *Sxl_Pe_* expression, the activities of heterochromatin proteins in dosage compensation appear to be distinct from the early roles of HP1 and HOAP at *Sxl_Pe_*. In addition, there may be zygotic roles for heterochromatin proteins in sex-specific gene expression, as proposed for HP1 by Liu et al. [41].

### Dynamic Roles of HOAP and HP1 at *Sxl_Pe_*

Previous analysis of *Sxl_Pe_* indicated that 400 bp immediately upstream of the promoter are sufficient for sex-specific regulation, but distal sequences, extending to −1700 bp, are required for wild type levels of expression [33]. As shown in Figure 4A, E-box binding sites for antagonistically acting bHLH proteins, which are encoded by zygotically expressed X-linked and autosomal signal elements (XSE and ASE) and direct an X counting mechanism, are distributed throughout both regions [49].

Both HP1 and HOAP are enriched in the region proximal to *Sxl_Pe_* which contains binding sites for both positive and negative E-box proteins. Within the *Sxl_Pe_* promoter distal region, HOAP alone is enriched in two peaks where there is a striking relationship with E-box binding sites for positive factors, but those for negative factors appear essentially devoid of HOAP. HOAP may antagonize positive factors but permit negative factors to bind in the *Sxl_Pe_* distal region, in an HP1-independent repressing role. Whereas loss of HOAP de-represses *Sxl_Pe_* in males, the strength and uniformity of expression does not approach that in wild type females. This indicates continued influence from the X counting mechanism in *cav* mutant males. *Sxl_Pe_* is also expressed prematurely in female embryos. This de-repression by reduced levels of maternal HOAP in both sexes indicates that HOAP is present at *Sxl_Pe_* in both sexes of wild type embryos. However, whether the proximal and distal *Sxl_Pe_* regions have the same or different compositions of HOAP and HP1 in the two sexes cannot be determined from our ChIP assays, as the embryos are of mixed sexual identity.

**Figure 6:**
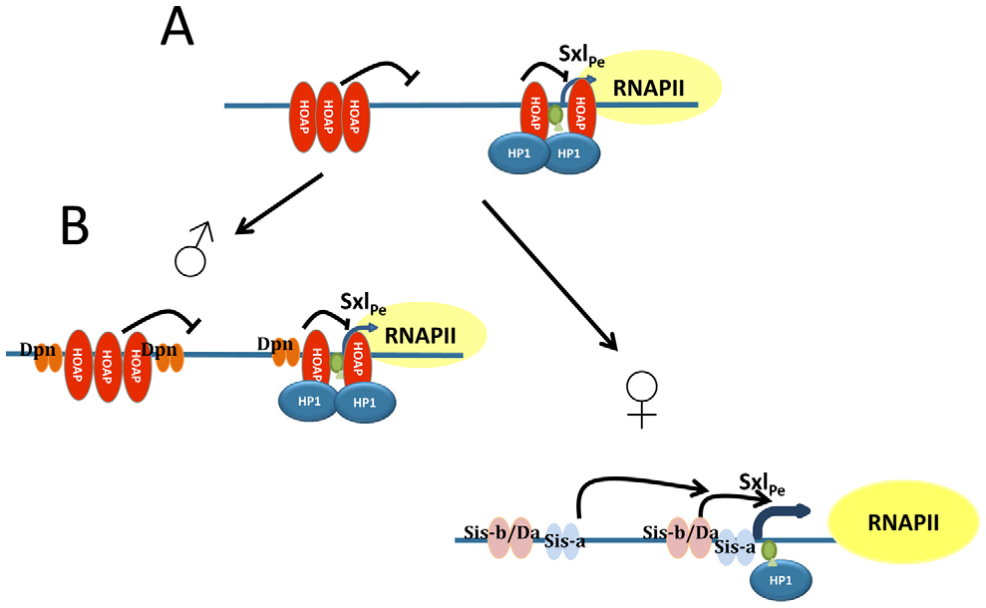
Model of the interactions between HOAP and HP1 at *Sxl_Pe_*. (A) Maternal HOAP and HP1 cooperate to form a repressive complex which serves to reduce the sensitivity of *Sxl_Pe_* to spurious fluctuations in zygotic expression of positive (e.g. Sis-a, Sis-b) and negative (e.g. Dpn) regulatory proteins. Pre-loading of RNA pol II requires HP1. (B) Binding of bHLH proteins to *Sxl_Pe_* sequences is dependent on their zygotic dose and binding sites relative to those for HOAP. At 2–3 hr of development, the two X chromosome dose of positive factors in females, is able to result in activation of *Sxl_Pe_*, and allow the pre-loaded RNA pol II to extend. In males, the single X chromosome presumably does not produce enough positive factors to displace (all of) the negative components. For simplicity not all known X∶A factors are shown. The triangle depicts low levels of H3K9 methylation at the promoter.

The interdependency of HOAP and HP1 for their binding to the *Sxl_P_*_e_ proximal region, most notably the dependence of HP1 on HOAP, also indicates both proteins are in this region in, at least, wild type female embryos. In spite of this interdependency, the genetic data show HOAP repression antagonizes HP1 activation. HOAP repression appears to also be partly HP1-dependent; the mutant HOAP protein from the *cav* ^1^ allele which lacks HP1-binding also antagonizes HP1 activation. This combination of antagonistic and cooperative interactions suggests a model in which maternal HOAP and HP1 first cooperate to repress *Sxl_Pe_* prior to its activation (Figure 6A). The repressive structure formed by maternal HOAP and HP1 likely serves to reduce the sensitivity of *Sxl_Pe_* to spurious fluctuations in zygotic XSE levels, ensuring it is only activated in females where an effective ratio of activating to repressing transcription factors exists (Figure 6B). HP1 is retained at *Sxl_Pe_* during its activation in females, where it presumably switches into an activation role. In early embryos constitutive heterochromatin proteins may be more appropriate for such regulation than the Polycomb Group of facultative heterochromatin proteins, as they would not be subject to cross regulatory signals from body plan specification pathways.

How HP1 switches over to transcriptional activation mode in the *Sxl*_Pe_ proximal region is unclear. Changes in HP1 phosphorylation and/or association with other factors could alter its activity [50], [51]. Several XSE binding sites are nearby, making them strong candidates. Presumably, this would only occur in females where the XSE dose surpasses a threshold and *Sxl_Pe_* is activated.

### Mechanism of HP1 in Transcriptional Activation at *Sxl_Pe_*

This report provides the most clearly defined role for HP1 in developmental control of a euchromatic gene in a metazoan species, and the first evidence of a bifunctional regulatory role for it in such a context. Prior reports describing HP1 in transcriptional activation have focused on it in the context of transcription elongation [11]–[13], [52], [53]. Our ChIP data at *Sxl_Pe_*, however, show a requirement of it for association of RNAP II with the promoter, more consistent with a role in transcription initiation. A role in initiation is also in keeping with the position of HP1 on the gene; we find very little HP1 elsewhere on the *Sxl* gene, even during the time of *Sxl_Pe_* activity. This dependence of RNAP II association on HP1 is similar to what is observed in the accumulation of noncoding RNAs at *S. pombe* centromeric repeats and mating type locus [54]. Nonetheless, it is possible that the loss of RNAP II at *Sxl_Pe_* reflects reduced stability of all RNAP II isoforms as a consequence of an early defect in transcription elongation, rather than a defect in RNAP II recruitment to the promoter.

Pausing of RNAP II in promoter proximal regions prior to activation has been observed in a high proportion of genes under developmental control in *Drosophila* embryos [55], and such pauses have also been implicated in regulation of alternative splicing [56]. While *Sxl_Pm_* appears to have the features of a promoter with paused RNAP II in a genome wide RNAP II ChIP study of 0–4 hr embryos (Flybase MODENCODE), RNAP II was absent from *Sxl_Pe_*. It is likely that the collection window for this study did not precisely coincide with the time of *Sxl_Pe_* activity. Our more narrowly timed collection indicates paused RNAP II at *Sxl_Pe_*, suggesting that, like *Sxl_Pm_*, it is a pre-loaded promoter. A preloaded *Sxl_Pe_* also readily explains how generalized up-regulation of phosphorylation of the RNAP II CTD by the loss of Nanos, causes *Sxl_Pe_* activation in males with an unchanged X∶A ratio [57].

### Evolutionary Implications of HOAP, Similarity to SRY?

Finally, the dominant negative activity of the *cav* ^2248^ allele suggests a role for the partially deleted SRY-like HMG box in HOAP association with *Sxl_Pe_*. Our ChIP data show HOAP association with the *Sxl_Pe_* proximal region is required for HP1 association. This proposed role for the HMG box of HOAP in *Sxl_Pe_* regulation is of particular interest with regards to a recent report linking HP1 and KAP-1 (TIF1β) to SRY-dependent repression of testis-specific genes in the ovary [26]. Because mammalian sex determination is inextricably linked to gonad sex determination, SRY and HOAP each appear to constitute early decision points in their respective sex determination pathways. There are, perhaps, unexpected parallels between these divergent pathways.

## Materials and Methods

### Genetic Analyses

The *cav* RNAi lines were obtained by germline transformation of the pUAST vector containing an inverted repeat of a near full length cDNA sequence for the *cav*-RB transcript (See Table S3 for primer sequences). The *cav* ^2248^ allele was recovered in a screen of progeny from ethane methyl sulfonate (EMS)-mutagenized males which failed to complement the original *cav* ^1^ allele, and the sequence of the allele was determined from PCR products obtained from homozygous *cav* ^2248^ embryos identified through their lack of the homologous twi-GAL4, UAS-GFP marked balancer chromosome.

Flies in all crosses were reared under uncrowded conditions on standard cornmeal medium enriched with active dry yeast. Unless otherwise noted, all crosses were done at 25°C; Ore R or *y* ^1^ *w* ^67c23^ were used as the wild type control. Progeny were counted for 8 days beginning on the first day of eclosion. The Z test was used in statistical analyses of distributions in two populations [58]. Description of genes can be found in Flybase (http://www.flybase.org/).

### Microarray Expression Profiling Study

RNA was TRIzol-extracted from *y* ^1^ *w* ^67c23^; *cav* ^1^ homozygous (those lacking the Tm6B,Tb balancer chromosome used to maintain the *cav* ^1^ mutation in heterozygous condition) and wild type first and second instar larvae and purified through Qiagen RNeasy columns. The RNA was then used by the University of Kentucky Microarray Facility to prepare biotin-labeled cRNA that was hybridized to separate Affymetrix *Drosophila* Genome microarrays (version 1). The *y* ^1^ *w* ^67c23^ stock to which all mutant stocks are out-crossed during genetic manipulations was used as the wild type control in these experiments. The data were obtained from two biological replicate samples from each stock. The statistical analyses of the arrays were carried out by A.J. Stromberg, PhD (UK Dept. of Statistics) associated with this facility according to standard Affymetrix specifications. Of 13,982 affytags to *Drosophila* genes on the array, an average of 46% and 53% were present in the *cav* ^1^ and wild type *y* ^1^ *w* ^67c23^ samples, respectively. Data from publically available tissue-specific cDNA libraries [35]–[37] and from two published microarray profiling studies of *Drosophila* sex- or tissue-specific gene expression [38], [39] were used to catalogue normal gene expression profiles of genes with reduced or elevated transcript levels (log_2_R>2.0, p<0.01).

### RT-PCR Analysis

RNA was similarly prepared from pools of male and female *cav* ^2248^ heterozygous and *cav* ^1^ homozygous embryos and from individual *Su(var)205* ^4^/*Su(var)205* ^5^ larvae. The *cav* ^2248^ heterozygous embryos were identified from a pool of tightly staged embryos that failed to progress to the larval stage and contained intermediate levels of GFP expression from the P{GAL4-twi.C}2.3, P{UAS-2xEGFP}AH2.3-marked TM3Sb balancer chromosome. The *cav* ^1^ homozygous embryos were similarly identified through their lack of GFP expression from this balancer chromosome. *Su(var)205* ^4^/*Su(var)205* ^5^ larvae were identified through their lack of GFP expression from the P{Act5C-GAL4}25F01-marked CyO balancer chromosome. The sex of individual *cav* ^2248^ heterozygous and *cav* ^1^ homozygous embryos was first determined through PCR of the Y-linked *kl-2* gene from a total nucleic acid extraction from individual embryos (See Table S3 for primer sequences). RNA was then purified from pools of nucleic acids from individual male or female embryos. The sex of individual *Su(var)205* ^4^/*Su(var)205* ^5^ larvae was first determined through the presence or absence of the *y* ^+^-marker from the paternally derived X chromosome and then substantiated through PCR assays of the Y-linked *kl-2* gene. RT-PCR assays were carried out with RNA purified from these pools using the Qiagen One-Step RT-PCR kit (Qiagen 210212). The sequences of all primers used are shown in Table S3. Primers for the *Drosophila RpA-70* were used in PCR reactions to determine that all RNA samples were DNA-free before they were used in RT-PCR as a positive control and in normalization of all RT-PCR data (Table S3).

### *In Situ* Hybridization

These were done as described in [59]. Digoxygenin labeled RNA probes complementary to *Sxl* exon E1 or L1 region were prepared using T7 RNA polymerase in vitro transcription of plasmid- or PCR-derived templates. The establishment (407 nt) and maintenance (1039 nt) transcript specific probes were generated by the primers shown in Table S3. All *in situs* were repeated at least once. Each batch was done simultaneously with an Ore R control and had sufficient embryos so that several representatives of each cycle could be examined.

### Chromatin Preparation

A modification of the protocol described in [60] was used to prepare cross-linked chromatin from embryonic progeny from parents of the following genotypes: wild type (*y* ^1^ *w* ^67c23^ or *yw*), *yw*; *cav* ^2248^/TM3Sb, *yw*; *Su(var)205* ^5^/CyO, *yw* females×*Sxl* ^f1^/Y males, and *yw*; *Su(var)205* ^5^/CyO females×*Sxl* ^f1^/Y males. Embryos from *yw* parents were collected at the following developmental stages: 0.75 to 2.75 hr (labeled 1–3 hr), 2–3 hr, and 4–18 hr. Embryos were collected from *yw*; *Su(var)205* ^5^/CyO females crossed to *Sxl* ^f1^/Y males in parallel to those from *yw* females crossed to *Sxl* ^f1^/Y males (at 2–3 hr stage). Embryos from *yw*; *cav* ^2248^/TM3Sb and from *yw*; *Su(var)205* ^5^/CyO parents were also collected in parallel (at 0.75 to 2.75 stage). All embryo collections and staging were done at 22°C. Chromatin was prepared from 6.0 g *yw* embryos in 6.0 ml homogenization buffer (50 mM Hepes at pH 7.6, 60 mM potassium chloride, 0.25 M sucrose, protease inhibitor cocktail [15]). The homogenate was first clarified by centrifugation at 500× g for 10 minutes before the addition of formaldehyde to 2%. Cross-linked chromatin was then washed 3 times in phosphate buffered saline (150 mM sodium chloride (NaCl), 10 mM sodium phosphate, pH 7.6) (with centrifugation at 3,000× g for 10 minutes after each wash) and re-suspended in 6.0 ml RIPA buffer (50 mM Tris-HCl, pH 7.6, 1 mM ethylene diamine tetraacetic acid (EDTA), 0.5 mM ethylene glycol tetraacetic acid (EGTA), 140 mM NaCl, 1% Triton X-100, 0.1% Na-deoxycholate, 0.1% SDS) and sonnicated to an average length of 500 bp. A scaled down version of the protocol was carried out with 0.5 g mutant embryos in 1.5 ml homogenization buffer.

### Chromatin Immunoprecipitations

Chromatin immunoprecipitations were performed with 0.2 ml clarified chromatin, 20–40 µg antibody [anti-HOAP [61], anti-HP1 [15], anti-RNA polymerase II antibody (Covance MMS-126R, MMS-134, or MMS-129R), anti-di and tri MeK9 histone H3 (Millipore 05-1249 and 05-1242) or non-immune IgG (Santa Cruz Biotech. SC-2027 or SC-2025)] and 100 µl anti-rabbit (Sigma A914) or anti-mouse (Sigma A6531) IgG agarose in 1.5 ml RIPA buffer. Washes were performed as described in Alekseyenko et al., [60]. The immunoprecipitated material was eluted from the beads by incubation at 37°C for 1 hr in 500 µl TE (1 mM EDTA, 50 mM Tris, pH 8.0) containing 0.5% sodium dodecyl sulfate (SDS) and proteinase K (0.1 mg/ml), followed by 12 hr at 65°C after the addition of NaCl to 0.3 M and SDS to 1%. The samples were then extracted once with phenol/chloroform, once with chloroform before ethanol precipitation in the presence of glycogen.

### Protocol for Quantitative Real-Time PCR

The iCycler iQ real-time PCR detection system (Bio Rad) was used to quantitate *Sxl* sequences in the precipitated DNA from each ChIP fraction. The primer pairs shown in Table S3 were used to amplify fragments spanning the *Sxl* locus as shown in Figure 4 and *RpA-70* normalizing standard (average length 288 bp). Similar enrichment values were obtained for all data when calculated as % of total in ChIP vs. input fraction. PCR amplification was performed in duplicate in 50 µl SYBR Green qPCR SuperMix (Bio-Rad 170–8880) on two biological replicates of each ChIP fraction. Dissociation curve analysis was performed at the end of 40 cycles, and quantification was carried out by Bio-Rad comparative C_T_ methodology with standard curves constructed for each primer pair with a serial dilution of input DNA having PCR efficiencies of 80–120%. A one sample t-test was performed to identify sequences that were enriched in ChIP fractions above background (i.e., >1); a student's t test was used to determine significance of differences between two samples of equal variance.

## Supporting Information

Figure S1 Characterizations of *cav* ^2248^ mutant embryos. (A) Cuticle preparations of genotyped *cav* ^2248^ homozygous and heterozygous embryos. (B) DAPI-staining of presumed pre-cycle 14 *cav* ^2248^ homozygous embryo and genotyped late-staged *cav* ^2248^ heterozygous embryo (Defects in *cav* ^2248^ homozygous embryos appear before expression of GFP marker used in genotyping.)
(TIF)

Figure S2 Representative PCR assays used to sex mutant animals. PCR assays of nucleic acids extracted from individual *cav* ^2248^ heterozygous (*cav* ^2248^/twi-GAL4, UAS-2x-EGFP TM3) or *cav* ^1^ homozygous (*cav* ^1^/*cav* ^1^) embryos or *Su(var)205* ^5^/*Su(var)205* ^4^ larvae using primers for *kl-2* Y-linked gene to sex animals and *RpA-70* as a positive control. Male embryos were identified as those yielding products with both sets of primers; female embryos were identified as those yielding products with *RpA-70* primers only. RNA was then isolated from pools of individually sexed *cav* mutant embryos or individual *Su(var)205* mutant larvae, and RT-PCR assays with *RpA-70* primers were used to assess the quality of each RNA template before using them in RT-PCR assays of *Sxl* transcripts.
(TIF)

Figure S3 *Sxl_Pm_* transcription is essentially unchanged in embryos from *cav^2248^*/TM3, Sb or *Su(var)205* ^5^/CyO parents. Comparisons of the same-sized area of images taken at 40×. Early to mid-cycle 14 female embryos from Ore R, *cav^2248^*/TM3 (*cav* ^2248^) or *Su(var)205* ^5^/CyO (*Su(var)205* ^5^) parents show essentially equivalent signal. Probe is specific to *Sxl_Pm_* transcripts, spanning exon 1 and small section of adjacent intron.
(TIF)

Table S1 Summary of tissue distribution of genes with decreased or increased transcript levels in *cav* ^1^ mutants.
(DOC)

Table S2 Categorization of genes with decreased or increased transcript levels in *cav* ^1^ mutant larvae. The normal tissue distribution of each gene set was assessed through a combination of data on its relative representation in tissue-specific cDNA libraries (cDNA Library Representation) [35], [36], [37]. Data of tissue-specific expression in adults by Chintapalli et al. [38] (tissue/adult enrichment) and sex-specific gonad expression by Parisi et al. [39] (testis/ovary enrichment).
(DOC)

Table S3 Oligonucleotides used as primers in this study.
(PDF)
