# Supplementary figures and images for: Cooperative and Antagonistic Contributions of Two Heterochromatin Proteins to Transcriptional Regulation of the Drosophila Sex Determination Decision
Source: PLoS Genet. 2011 Jun 9;7(6):e1002122. doi: 10.1371/journal.pgen.1002122 (PMC3111545; doi:10.1371/journal.pgen.1002122)

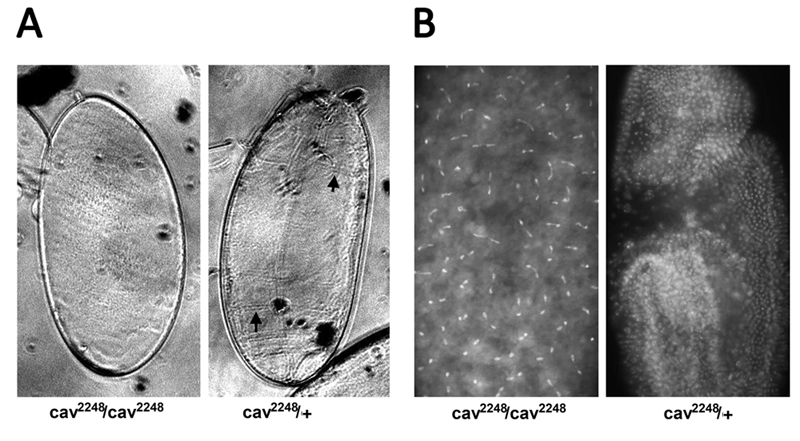

Supplement: Figure S1 — Characterizations of cav 2248 mutant embryos. (A) Cuticle preparations of genotyped cav 2248 homozygous and heterozygous embryos. (B) DAPI-staining of presumed pre-cycle 14 cav 2248 homozygous embryo and genotyped late-staged cav 2248 heterozygous embryo (Defects in cav 2248 homozygous embryos appear before expression of GFP marker used in genotyping.) (TIF) [file pgen.1002122.s001.tif]

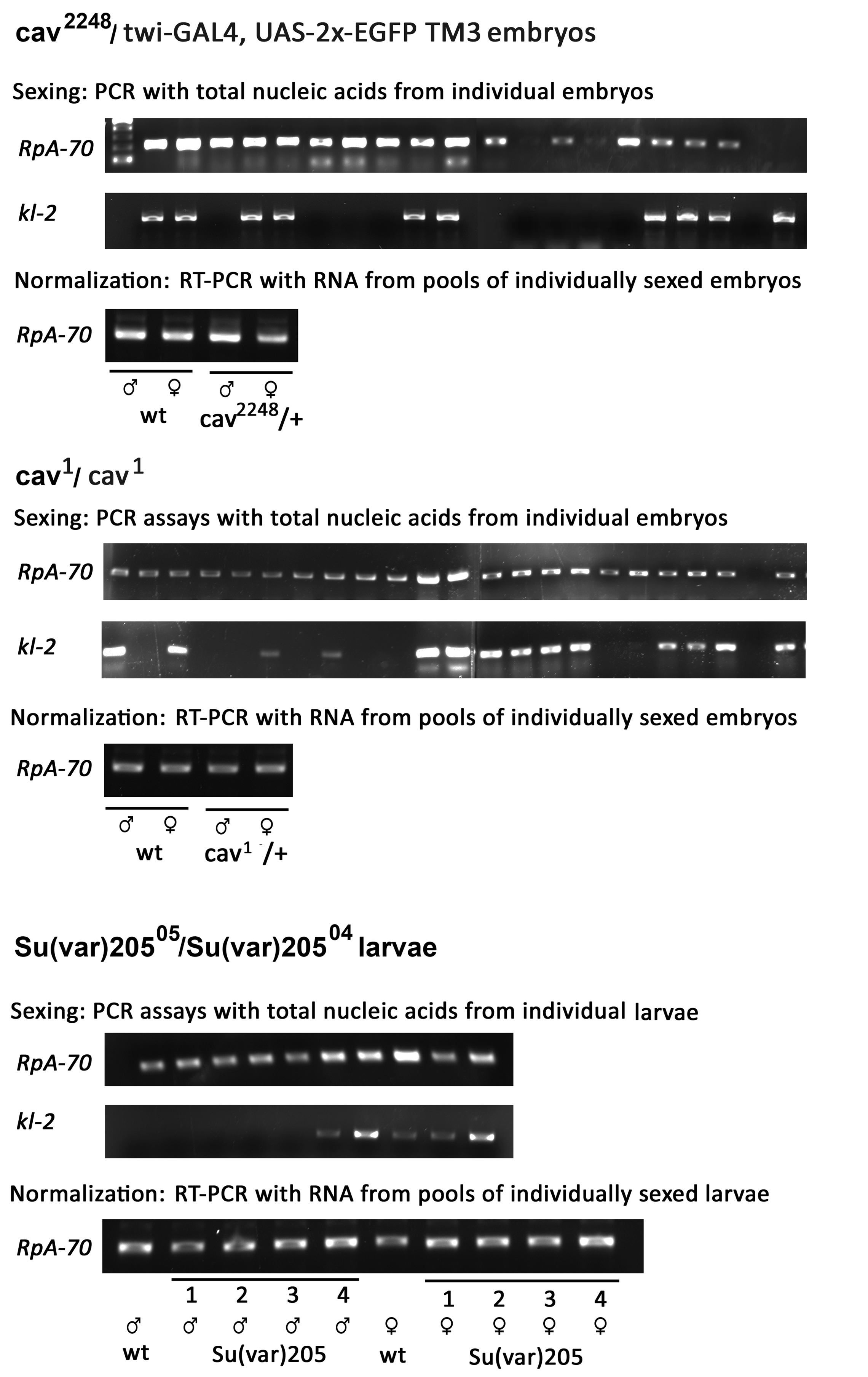

Supplement: Figure S2 — Representative PCR assays used to sex mutant animals. PCR assays of nucleic acids extracted from individual cav 2248 heterozygous (cav 2248/twi-GAL4, UAS-2x-EGFP TM3) or cav 1 homozygous (cav 1/cav 1) embryos or Su(var)205 5/Su(var)205 4 larvae using primers for kl-2 Y-linked gene to sex animals and RpA-70 as a positive control. Male embryos were identified as those yielding products with both sets of primers; female embryos were identified as those yielding products with RpA-70 primers only. RNA was then isolated from pools of individually sexed cav mutant embryos or individual Su(var)205 mutant larvae, and RT-PCR assays with RpA-70 primers were used to assess the quality of each RNA template before using them in RT-PCR assays of Sxl transcripts. (TIF) [file pgen.1002122.s002.tif]

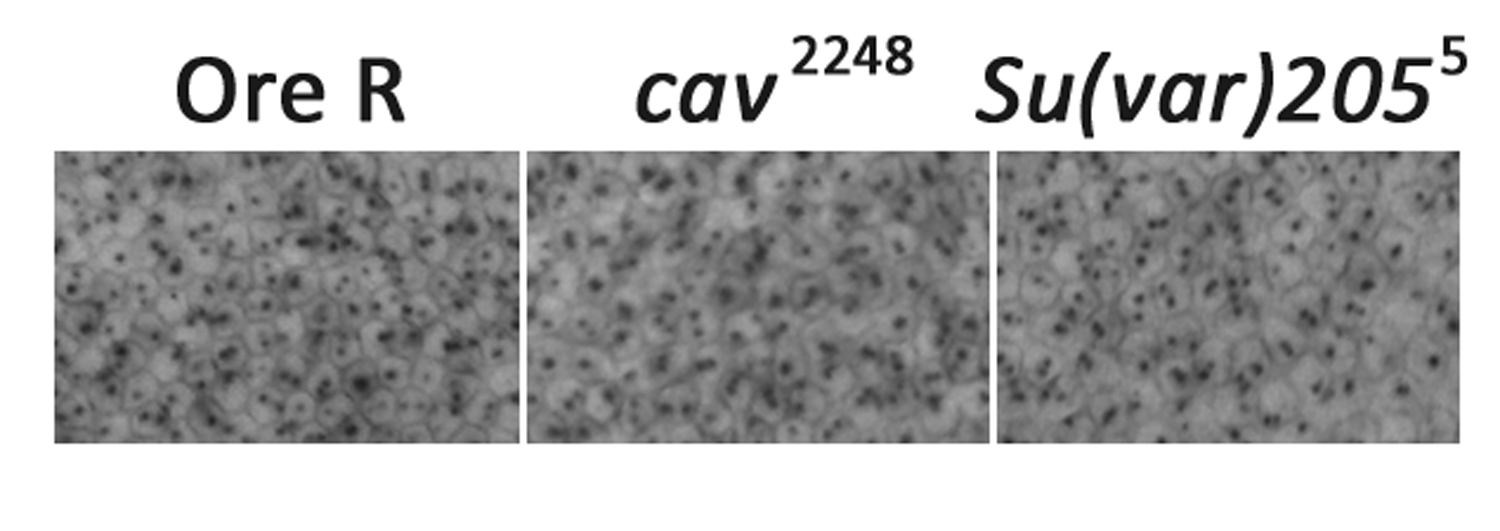

Supplement: Figure S3 — SxlPm transcription is essentially unchanged in embryos from cav2248/TM3, Sb or Su(var)205 5/CyO parents. Comparisons of the same-sized area of images taken at 40×. Early to mid-cycle 14 female embryos from Ore R, cav2248/TM3 (cav 2248) or Su(var)205 5/CyO (Su(var)205 5) parents show essentially equivalent signal. Probe is specific to SxlPm transcripts, spanning exon 1 and small section of adjacent intron. (TIF) [file pgen.1002122.s003.tif]
