# Supplementary material for: Cooperative and Antagonistic Contributions of Two Heterochromatin Proteins to Transcriptional Regulation of the Drosophila Sex Determination Decision
Source: PLoS Genet. 2011 Jun 9;7(6):e1002122. doi: 10.1371/journal.pgen.1002122 (PMC3111545; doi:10.1371/journal.pgen.1002122)
Supplement: Table S1 — Summary of tissue distribution of genes with decreased or increased transcript levels in cav 1 mutants. (DOC) [file pgen.1002122.s004.doc]

**Table S1**

**Genes with Reduced Transcript Levels: Genes with Elevated Transcript Levels:**

**Tissue # Genes Fraction Tissue # Genes Fraction**

Embryo: 1 0.7% Carcass: 1 2.5%

Multiple: 34 23.9% Midgut: 8 19.5%

Ovary: 4 2.8% Multiple: 24 58.5%

Rare: 4 2.8% Rare: 8 19.5%

Testis: 95 67.0% Total: 41 100%

Testis & Ovary: 4 2.8%

Total: 142 100%
