# Supplementary material for: Cooperative and Antagonistic Contributions of Two Heterochromatin Proteins to Transcriptional Regulation of the Drosophila Sex Determination Decision
Source: PLoS Genet. 2011 Jun 9;7(6):e1002122. doi: 10.1371/journal.pgen.1002122 (PMC3111545; doi:10.1371/journal.pgen.1002122)
Supplement: Table S2 — Categorization of genes with decreased or increased transcript levels in cav 1 mutant larvae. The normal tissue distribution of each gene set was assessed through a combination of data on its relative representation in tissue-specific cDNA libraries (cDNA Library Representation) [35], [36], [37]. Data of tissue-specific expression in adults by Chintapalli et al. [38] (tissue/adult enrichment) and sex-specific gonad expression by Parisi et al. [39] (testis/ovary enrichment). (DOC) [file pgen.1002122.s005.doc]

**Table S2**

| **Affy Tag- Drosophila**  **Genome 1** | **log 2 R** | **p value** | **Gene**  **Name** | **cDNA Library**  **Representation**  **[36,37,38]** | **Chintapalli**  **et al. [39] tissue/adult**  **enrichment** | **Parisi et al. [40]**  **testes/ovary enrichment** | **Compiled Expression**  **Data** |
| --- | --- | --- | --- | --- | --- | --- | --- |
| ***Genes with Reduced Transcript Levels*** | | | | | | | |
| 145319_at | -2.08 | 0.003 | phm | embryo | none | N/A | embryo |
| 148103_at | -69.61 | 0.004 | dro5 | multiple | multiple | N/A | multiple |
| 141293_at | -33.65 | 0.009 | CG9362 | multiple | multiple | N/A | multiple |
| 141440_at | -14.13 | 0.01 | b | multiple | multiple | N/A | multiple |
| 153219_at | -12.12 | 0.004 | CG7768 | multiple | multiple | N/A | multiple |
| 143559_at | -7.91 | 0.008 | ple | multiple | multiple | N/A | multiple |
| 143970_at | -7.34 | 0.002 | Clk | multiple | multiple | N/A | multiple |
| 144311_at | -6.78 | 0.006 | CG4180 | multiple | multiple | N/A | multiple |
| 142188_at | -5.9 | 0.003 | Pif1b | multiple | multiple | N/A | multiple |
| 152004_at | -5.52 | 0.009 | eya | multiple | multiple | N/A | multiple |
| 143403_at | -5.55 | 0.006 | vg | multiple | multiple | N/A | multiple |
| 149054_at | -5.01 | 0.006 | CG9452 | rare | multiple-low | N/A | multiple |
| 155099_at | -4.30 | 0.003 | bib | multiple | multiple | N/A | multiple |
| 153488_at | -3.45 | 0.001 | heph | multiple | multiple | N/A | multiple |
| 152034_at | -3.34 | 0.008 | CG10139 | multiple | multiple | N/A | multiple |
| 152265_at | -3.30 | 0.008 | CG11892 | multiple | multiple | N/A | multiple |
| 150844_at | -3.19 | 0.009 | CG12068 | multiple | multiple-low | N/A | multiple |
| 155062_at | -3.18 | 0.01 | BEAF-32 | multiple | multiple | N/A | multiple |
| 149779_at | -3.12 | 0.004 | CG18549 | multiple | multiple | N/A | multiple |
| 143268_at | -3.09 | 0.01 | mnd | multiple | multiple | N/A | multiple |
| 144536_at | -3.01 | 0 | Tre1 | multiple | multiple | N/A | multiple |
| 145677_at | -3 | 0.007 | CG3117 | rare | multiple | N/A | multiple |
| 154844_at | -2.79 | 0.009 | cdc2 | multiple | multiple | N/A | multiple |
| 143464_f_at | -2.78 | 0.008 | LysB | multiple | N/A | N/A | multiple |
| 144734_at | -2.71 | 0.002 | CG12115 | multiple | multiple | N/A | multiple |
| 153480_at | -2.69 | 0.009 | Map60 | multiple | multiple | N/A | multiple |
| 141388_at | -2.64 | 0.009 | dap | multiple | multiple | N/A | multiple |
| 150195_at | -2.64 | 0.01 | CG5316 | multiple | multiple | N/A | multiple |
| 146874_at | -2.47 | 0.003 | CG2063 | multiple | multiple | N/A | multiple |
| 145098_at | -2.43 | 0.007 | Lsd-2 | multiple | multiple | N/A | multiple |
| 142578_at | -2.28 | 0.002 | pr-set7 | multiple | multiple | N/A | multiple |
| 154656_at | -2.15 | 0.009 | Aos1 | multiple | multiple | N/A | multiple |
| 153930_at | -2.13 | 0.008 | CG5224 | multiple | multiple | N/A | multiple |
| 153243_at | -2.11 | 0.002 | lwr | multiple | multiple | N/A | multiple |
| 151082_at | -2.08 | 0.008 | CG33111 | multiple | multiple | testis (6.8x) | multiple |
| 143679_at | -2.06 | 0.005 | spn-A | multiple | ovary (1.8x) | N/A | ovary |
| 149186_at | -2.28 | 0.005 | Mkrn1 | multiple | ovary (2.3x) | N/A | ovary |
| 143834_at | -2.26 | 0.008 | CycB3 | multiple | ovary (2.3x) | N/A | ovary |
| 141239_at | -2.22 | 0.009 | ovo | rare | ovary (2.9x) | ovary | ovary |
| 141337_at | -53.44 | 0.006 | CG7906 | rare | eye (113x) | N/A | rare |
| 143465_f_at | -11.52 | 0.006 | LysC | rare | N/A | N/A | rare |
| 145643_at | -8.16 | 0.001 | CG31684 | rare | none | N/A | rare |
| 145734_at | -4.84 | 0.002 | CG2816 | rare | multiple-low | N/A | rare |
| 152219_at | -22.03 | 0.006 | CG6304 | testis (57%) | testis (9.9x) | testis(29.7x) | ***testis*** |
| 148801_at | -20.31 | 0.009 | CG7804 | testis | testis (14.8x) | N/A | ***testis*** |
| 149981_at | -19.92 | 0.003 | CG4546 | testis (46%) | testis (10.0x) | testis (10.8x) | ***testis*** |
| 145909_at | -18.45 | 0.007 | CG11043 | testis | testis (10.3x) | testis (6.4x) | ***testis*** |
| 152703_at | -17.93 | 0.003 | CG7848 | testis (60%) | testis (12.2x) | testis (11.0x) | ***testis*** |
| 142968_at | -17.84 | 0.01 | CG9279 | testis (58%) | testis (6.5x) | testis (37.2x) | ***testis*** |
| 144854_at | -17.03 | 0.001 | CG15200 | testis | testis (10.4x) | testis (23.0x) | ***testis*** |
| 142673_at | -14.42 | 0 | CG8292 | testis | testis (15.3x) | testis (16.1x) | ***testis*** |
| 150757_at | -14.12 | 0.009 | CG5017 | testis | testis (8.9x) | testis (19.3x) | ***testis*** |
| 147290_at | -13.97 | 0.01 | CG3687 | testis | testis (12.5x) | testis (9.0x) | ***testis*** |
| 152992_at | -13.46 | 0.007 | CG10934 | testis (50%) | testis (13.9x) | N/A | ***testis*** |
| 146671_at | -13.39 | 0 | CG14589 | rare | testis (10.2x) | testis (5.1x) | ***testis*** |
| 152628_at | -12.86 | 0.004 | CG1999 | rare | testis (10.4x) | testis (16.2x) | ***testis*** |
| 142331_i_at | -12.63 | 0.002 | CG3875 | testis | testis (14.2x) | N/A | ***testis*** |
| 144514_at | -12.32 | 0.009 | TrxT | N/A | testis (6.3x) | testis (19.9x) | ***testis*** |
| 150334_at | -11.69 | 0.004 | CG31178 | testis | testis (10.4x) | testis (47.3x) | ***testis*** |
| 146743_at | -11.67 | 0.005 | CG11125 | testis | testis (11.9x) | testis (10.8x) | ***testis*** |
| 151006_at | -11.26 | 0.006 | CG15219 | testis (71%) | testis (3.4 x) | testis (4.5x) | ***testis*** |
| 147441_at | -11.05 | 0.001 | CG17669 | testis | testis (14.4x) | testis (5.9x) | ***testis*** |
| 148940_at | -10.82 | 0.003 | CG13725 | testis | testis (10.2x) | testis (9.7x) | ***testis*** |
| 151765_at | -10.43 | 0.007 | CG31624 | testis | testis (4.4x) | testis (34.6x) | ***testis*** |
| 146412_at | -10.2 | 0.009 | CG7094 | testis | testis (13.2x) | testis (8.0x) | ***testis*** |
| 144885_at | -9.74 | 0.01 | CG11697 | testis | testis (13.8x) | testis (8.3x) | ***testis*** |
| 149928_at | -9.71 | 0.01 | RpL10Aa | testis | testis (9.2x) | testis (34.7x) | ***testis*** |
| 144120_at | -9.27 | 0.002 | CkIIbeta2 | testis | testis (13.1x) | testis (16.0x) | ***testis*** |
| 143393_at | -9.26 | 0.01 | betaTub85D | testis (77%) | testis (5.6x) | testis (33.5) | ***testis*** |
| 146804_at | -9.19 | 0.009 | CG18449 | testis | testis (6.0x) | testis (31.2) | ***testis*** |
| 146302_at | -9.14 | 0.006 | ACXA | testis | testis (20.1x) | testis (6.9x) | ***testis*** |
| 147634_at | -9.03 | 0 | Grx-1 | testis | testis (14.6x) | testis (7.2x) | ***testis*** |
| 150197_at | -8.76 | 0.008 | CG3517 | testis (90%) | testis (8.3x) | testis (34.0x) | ***testis*** |
| 153056_at | -8.29 | 0.009 | ssp5 | testis (98%) | testis (9.7x) | testis (58.8x) | ***testis*** |
| 146241_at | -8.26 | 0.008 | CG14926 | testis (79%) | testis (4.8x) | testis (43.2x) | ***testis*** |
| 152333_at | -8.17 | 0.002 | sm | testis (50%) | testis (7.1x) | testis (29.6x) | ***testis*** |
| 147684_f_at | -7.89 | 0.004 | CG3927 | testis | testis (13.6x) | testis (5.9x) | ***testis*** |
| 147518_at | -7.56 | 0.01 | CG8517 | testis | testis (6.8x) | testis (22.3x) | ***testis*** |
| 151443_at | -7.5 | 0.004 | CG13245 | testis (71%) | testis (5.7x) | N/A | ***testis*** |
| 144286_at | -7.36 | 0.008 | CG4218 | testis | testis (10.8x) | N/A | ***testis*** |
| 151906_at | -7.33 | 0.004 | CG5538 | testis (73%) | testis (8.1x) | testis (39.1x) | ***testis*** |
| 145981_at | -7.1 | 0.001 | CG7196 | testis | testis (12.9) | testis (12.9x) | ***testis*** |
| 143686_at | -7.06 | 0.001 | Arp53D | testis | testis (14.2) | N/A | ***testis*** |
| 143432_at | -6.81 | 0.01 | Mst98Cb | N/A | testis (7.2x) | testis (90.5x) | ***testis*** |
| 146857_at | -6.8 | 0.001 | CG13747 | testis | testis (10.1x) | testis (4.7x) | ***testis*** |
| 146552_at | -6.79 | 0.008 | CG33322 | testis | testis (16.6) | N/A | ***testis*** |
| 144488_at | -6.78 | 0.009 | CG7024 | testis | testis (9.8x) | testis (12.6x) | ***testis*** |
| 143434_at | -6.74 | 0.01 | Mst84Dd | testis | testis (4.2x) | testis (63.9x) | ***testis*** |
| 151334_at | -6.71 | 0.004 | CR32658 | testis | N/A | testis (16.2x) | ***testis*** |
| 150336_at | -6.63 | 0.003 | CG17819 | testis (67%) | testis (15.5x) | N/A | ***testis*** |
| 145709_at | -6.4 | 0.009 | CG8840 | testis | testis (10.6x) | testis (13.0x) | ***testis*** |
| 145439_at | -6.23 | 0.009 | hydra | testis | testis (10.2x) | N/A | ***testis*** |
| 149662_at | -6.22 | 0.001 | CG6629 | testis | testis (10.9x) | testis (29.9x) | ***testis*** |
| 145156_at | -6.11 | 0.006 | CG8565 | N/A | testis (11.8x) | testis (40.2x) | ***testis*** |
| 150454_at | -6.1 | 0.005 | CG10164 | testis | testis (11.0x) | N/A | ***testis*** |
| 149978_at | -6.08 | 0.006 | CG14876 | testis | testis (12.8x) | N/A | ***testis*** |
| 146777_at | -6.03 | 0.001 | CG30378 | testis | testis (10.8x) | N/A | ***testis*** |
| 145607_at | -5.92 | 0.005 | CG7295 | testis | testis (14.3x) | testis (8.3x) | ***testis*** |
| 141371_at | -5.78 | 0 | CG13263 | testis | testis (8.3x) | N/A | ***testis*** |
| 152084_at | -5.7 | 0.003 | sut4 | testis | testis (11.9x) | N/A | ***testis*** |
| 152092_at | -5.4 | 0.005 | CG4323 | testis | testis (11.9x) | testis (28.0x) | ***testis*** |
| 146081_at | -5.39 | 0.003 | CG13110 | testis | testis (12.1x) | testis (11.0x) | ***testis*** |
| 153027_at | -5.29 | 0.006 | CG4669 | testis (88%) | testis (7.8x) | testis (56.4x) | ***testis*** |
| 145978_at | -5.23 | 0.007 | CG7211 | testis | testis (9.4x) | N/A | ***testis*** |
| 143795_at | -5.22 | 0.001 | Ssl | testis | testis (11.6x) | N/A | ***testis*** |
| 152391_at | -4.89 | 0.001 | CG1340 | testis (78%) | testis (16.4x) | N/A | ***testis*** |
| 152731_at | -4.79 | 0.009 | CG8838 | testis | testis (8.9x) | testis (34.1) | ***testis*** |
| 151140_at | -4.74 | 0.005 | CG30222 | testis | testis (10.6x) | testis(12.8x) | ***testis*** |
| 146805_at | -4.72 | 0.005 | CG2127 | testis | testis (3.8x) | testis (106x) | ***testis*** |
| 145464_at | -4.62 | 0.003 | CG14579 | testis | testis (16.3x) | testis (3.0x) | ***testis*** |
| 148475_at | -4.55 | 0.008 | CG3222 | testis | testis (10.9x) | testis (23.9x) | ***testis*** |
| 151971_at | -4.51 | 0.006 | CG11060 | testis | testis (8.1x) | N/A | ***testis*** |
| 148765_at | -4.33 | 0.008 | CG13471 | testis | testis (12.5x) | N/A | ***testis*** |
| 152645_at | -4.32 | 0.006 | CG7742 | testis | testis (9.7x) | testis (21.6x) | ***testis*** |
| 144447_at | -4.27 | 0.009 | CG13021 | testis | testis (5.0x) | N/A | ***testis*** |
| 143274_at | -4.27 | 0.003 | Mst87F | testis | testis (2.6x) | testis (49.1x) | ***testis*** |
| 151454_at | -4.2 | 0.003 | CG12167 | testis | testis (4.3x) | N/A | ***testis*** |
| 150449_at | -4.2 | 0 | CG10177 | testis | testis (17.2x) | testis (37.5x) | ***testis*** |
| 143725_at | -4.06 | 0.004 | Dhc36C | testis | testis (9.5x) | testis (4.1x) | ***testis*** |
| 142792_at | -4.04 | 0.007 | CG11386 | rare | testis (14.5x) | N/A | ***testis*** |
| 152890_at | -3.72 | 0.007 | CG10126 | testis | testis (4.5x) | N/A | ***testis*** |
| 144811_at | -3.65 | 0.003 | CG15296 | testis | testis (10.8x) | testis (6.9x) | ***testis*** |
| 150362_at | -3.65 | 0.001 | CG13855 | testis | testis (11.7x) | N/A | ***testis*** |
| 147932_at | -3.58 | 0 | Ppm1 | testis | testis (12.5x) | testis (10.0x) | ***testis*** |
| 151537_at | -3.55 | 0.008 | CG33278 | testis | testis (19.1x) | N/A | ***testis*** |
| 147858_at | -3.52 | 0.003 | CG3494 | testis | testis (10.2x) | testis (18.6x) | ***testis*** |
| 146608_at | -3.33 | 0.003 | CG10834 | rare | testis (17.9x) | N/A | ***testis*** |
| 150618_at | -3.27 | 0.001 | CG17770 | testis | testis (12.9x) | testis (23.1x) | ***testis*** |
| 152716_at | -3.24 | 0.01 | CG3492 | testis | testis (11.0x) | testis (22.5x) | ***testis*** |
| 149430_at | -3.16 | 0.008 | CG1288 | testis | N/A | testis (33.6x) | ***testis*** |
| 145315_at | -3.09 | 0.01 | CG15040 | rare | testis (9.1x) | N/A | ***testis*** |
| 147667_at | -3.01 | 0.008 | CG11291 | testis | testis (14.1x) | N/A | ***testis*** |
| 150038_at | -2.72 | 0.005 | CG14909 | rare | testis (12.0x) | testis (5.7x) | ***testis*** |
| 148908_at | -2.7 | 0.009 | CG13032 | testis | testis (13.0x) | N/A | ***testis*** |
| 146602_at | -2.59 | 0.004 | CG1421 | N/A | testis (14.5x) | testis (3.6x) | ***testis*** |
| 146245_at | -2.48 | 0.004 | CG14930 | testis | testis (15.1x) | N/A | ***testis*** |
| 142311_at | -2.41 | 0.005 | Ubc84D | testis | testis (12.7x) | testis (13.8x) | ***testis*** |
| 149858_at | -2.13 | 0.006 | CG9602 | testis | testis (9.8x) | testis (10.4x) | ***testis*** |
| 154179_at | -6.01 | 0.005 | CG8478 | multiple | testis, ovary (2.8x) | N/A | testis/ovary |
| 149176_at | -4.32 | 0.001 | E(bx) | testis | testis, ovary (2.0x) | N/A | testis/ovary |
| 147842_at | -2.57 | 0.004 | spag | testis | testis, ovary (2.0x) | N/A | testis/ovary |
| 153420_at | -2.54 | 0.007 | Neos | multiple | testis, ovary (2.0x) | N/A | testis/ovary |
| 141299_at | -2.03 | 0.009 | CG12104 | embryo | testis, ovary (3.0x) | N/A | testis/ovary |
| ***Genes with Elevated Transcript Levels*** | | | | | | | |
| 151237_at | 8.17 | 0.006 | CG13230 | none | carcass | N/A | carcass |
| 142147_at | 3.01 | 0.009 | CG9466 | multiple | midgut (7.5x) | N/A | midgut |
| 141389_at | 6.85 | 0.009 | Cht9 | ubiq | midgut (20.0x) | N/A | midgut |
| 144327_at | 8.48 | 0.002 | CG7631 | rare | midgut (12.5x) | N/A | midgut |
| 147334_at | 9.83 | 0.006 | CG5550 | rare | midgut (12.5x) | N/A | midgut |
| 143695_at | 10.25 | 0.004 | pcl | rare | midgut (17.5x) | N/A | midgut |
| 145842_at | 10.32 | 0.008 | CG11149 | rare | midgut (20.0x) | N/A | midgut |
| 142359_at | 15.42 | 0.004 | CG30360 | rare | midgut (15.6x) | N/A | midgut |
| 147598_at | 21.43 | 0.005 | Cht4 | rare | midgut (11.5x) | N/A | midgut |
| 147498_at | 2.09 | 0 | CalpA | multiple | multiple | N/A | multiple |
| 151429_at | 2.11 | 0.001 | CG14933 | multiple | multiple | N/A | multiple |
| 153172_at | 2.18 | 0.008 | CG31886 | multiple | multiple | N/A | multiple |
| 152413_at | 2.2 | 0.006 | l(2)k05819 | multiple | multiple | N/A | multiple |
| 147171_at | 2.31 | 0.007 | CG6337 | multiple | multiple | N/A | multiple |
| 154652_at | 2.32 | 0.006 | alpha-Est1 | multiple | multiple | N/A | multiple |
| 149632_at | 2.51 | 0.006 | Cyp12e1 | multiple | multiple | N/A | multiple |
| 149722_at | 3.07 | 0.008 | CG18547 | rare | multiple | N/A | multiple |
| 144407_at | 2.54 | 0.003 | CG8310 | rare | multiple | N/A | multiple |
| 145813_at | 2.64 | 0.008 | Cyp28d2 | multiple | multiple | N/A | multiple |
| 141351_at | 2.8 | 0.007 | kst | multiple | multiple | N/A | multiple |
| 152848_at | 2.85 | 0.001 | CG1698 | multiple | multiple | N/A | multiple |
| 147607_at | 3.02 | 0.007 | Glycogenin | multiple | multiple | N/A | multiple |
| 142254_at | 3.18 | 0.007 | CG10562 | multiple | multiple | N/A | multiple |
| 154737_at | 3.31 | 0 | Ndg | multiple | multiple | N/A | multiple |
| 153087_at | 3.57 | 0.008 | CG8654 | multiple | multiple | N/A | multiple |
| 142969_at | 3.82 | 0.005 | Cys | multiple | multiple | N/A | multiple |
| 144364_at | 4.32 | 0.01 | CG14630 | multiple | multiple | N/A | multiple |
| 152083_at | 4.45 | 0.006 | CG15093 | multiple | multiple | N/A | multiple |
| 149937_at | 4.55 | 0.005 | CG6912 | multiple | multiple | N/A | multiple |
| 152779_at | 6.19 | 0.004 | CG8774 | multiple | multiple | N/A | multiple |
| 149964_at | 6.35 | 0.01 | CG14872 | rare | multiple | N/A | multiple |
| 142746_at | 6.77 | 0.003 | Men | multiple | multiple | N/A | multiple |
| 145897_at | 18.05 | 0.001 | CG42369 | multiple | multiple | N/A | multiple |
| 149889_at | 2.05 | 0.005 | CG3259 | rare | multiple-low | N/A | rare |
| 145058_at | 2.65 | 0.004 | CG1461 | none | multiple | N/A | rare |
| 153504_at | 3.23 | 0.009 | CG33137 | none | multiple-low | N/A | rare |
| 147127_s_at | 3.56 | 0.002 | CG13323 | rare | multiple-low | N/A | rare |
| 150252_at | 4.27 | 0 | CG4362 | rare | multiple-low | N/A | rare |
| 154595_at | 4.65 | 0.002 | CG7526 | rare | multiple | N/A | rare |
| 147126_i_at | 5.63 | 0.001 | CG13323 | rare | multiple | N/A | rare |
| 148709_at | 5.88 | 0.002 | CG14105 | rare | multiple-low | N/A | rare |
