# Supplementary material for: Cooperative and Antagonistic Contributions of Two Heterochromatin Proteins to Transcriptional Regulation of the Drosophila Sex Determination Decision
Source: PLoS Genet. 2011 Jun 9;7(6):e1002122. doi: 10.1371/journal.pgen.1002122 (PMC3111545; doi:10.1371/journal.pgen.1002122)
Supplement: Table S3 — Oligonucleotides used as primers in this study. (PDF) [file pgen.1002122.s006.pdf]

	Sequence	GadFly Coordinates
**PCR frag in Fig. 4:**		
+7101F	CAGCTCGCGATCGGTCATGT	6979712-6979731
+7101R	CGGCGGAAATGGTGGGAGTG	6979916-6979897
+1218F	GCCAAAGAGGTATGGGTAGC	6985512-6985531
+1218R	GTGGTTATCCCCCATATGGC	6985743-6985724
+138F	CGCTTATCCAGGGTTGCCATACCA	6986594-6986617
+138R	CGGCACCCCTCTGTCGATCC	6986960-6986941
-101F	GTCCTGCGGGCCATCGATCT	6986915-6986934
-101R	CGGGGATCCGCGAAATGCAG	6987117-6987098
-432F	GCCGAAAGGTGGAACGTGGC	6987225-6987244
-432R	GTATTGTGCTGGTATTGCTTC	6987469-6987449
-918F	TTTTGCTAGGATTTCGGGTAG	6987721-6987741
-918R	CTGTTCTTGGCGTTCGATTAG	6987944-6987924
-1132F	CTAATCGAACGCCAAGAACAG	6987924-6987944
-1132R	CTAGGGCTTAGGGCTCACTGG	6988169-6988149
-1394F	TTCCAGTGAGCCCTAAGCCCTAGCC	6988147-6988171
-1394R	TAGGCCGCCCCACCCACTTG	6988463-6988444
-1810F	GCTGGAATGGGGTGGTACGG	6988510-6988529
-1810R	CCCCAAAAGTTTTGTCCGTGATGGAC	6988940-6988915
-2224F	GTCCATCACGGACAAAACTTTTGGGG	6988915-6988940
-2224R	AGAACACACATTCACTCAAGCACTGC	6989362-6989337
-2647F	GATGCATGTACACATCTGTGC	6989424-6989444
-2647R	ATATGTTTCGGTTCCGCAATG	6989719-6989699
-3488F	GAAGCGAACATCTTCCCTTGC	6990249-6990269
-3488R	CGACCGCCCTACGTCGACGGC	6990558-6990538
-5094F	ACACGGATTGCATGGGGCACA	6991909-6991929
-5094R	TGACCGACAACCGCGAAACGTG	6992109-6992088
-5331F	CACGTTTCGCGGTTGTCGGTCA	6992088-6992109
-5331R	TGAACGAAATCTAACGGGGCTCAAAAGAC	6992404-6992376
***Sxl* RT-PCR:**		
*SxlP1*F	CACCGCTGCCCAGCGACAAT	6979801-6979820
*SxlP2*F	TTGCGTTTCGTTGGCGAGGACC	6982643-6982662
*SxlP3*R	GAGCGCTGAGCGCCAAAACAATTGA	6985955-6985931
***Sxl in situs*:**		
SxlPeF	GTTTCTAAGCAGATCCCG	6986495-6986512
Sxl PeR	GTTCCACTCGTGACAAGTCC	6986902-6986883
SxlPmF	GGGCGATGCTTGCATGTTGC	6991059-6991078
SxlPmR	GCGAAACGTGCACACTGC	6992097-6992080
**ChIP control primers:**		
*RpA70*F	CACCGCCGAGGCTCAAGGAC	4088834-4088853
*RpA70*R	TGCAAGGAGGTGGGCGAGTTG	4089056-4089036
*hb*F	GCCTGTCAATGCTGGCGACTTTCG	4523493-4523516
*hb*R	GAAAGAGCGAACGCCACGAAGGG	4523867-4523845
*kl-2*F	GGTTGCCCAATGTGAGTGAAGCGTC	82313-82337
*kl-2*R	TCGCTTAGCTCGCCGCCGTA	82775-82756
